# The current status of resistance to alpha-cypermethrin, ivermectin, and amitraz of the cattle tick (*Rhipicephalus microplus*) in Ecuador

**DOI:** 10.1371/journal.pone.0174652

**Published:** 2017-04-07

**Authors:** Richar Rodríguez-Hidalgo, Ximena Pérez-Otáñez, Sandra Garcés-Carrera, Sophie O. Vanwambeke, Maxime Madder, Washington Benítez-Ortiz

**Affiliations:** 1 Instituto de Investigación en Salud Pública y Zoonosis / Universidad Central del Ecuador, Quito, Ecuador; 2 Facultad de Medicina Veterinaria y Zootecnia / Universidad Central del Ecuador, Quito, Ecuador; 3 Instituto Nacional de Investigaciones Agropecuarias, Quito, Ecuador; 4 Georges Lemaître Centre for Earth and Climate research, Université Catholique de Louvain, Louvain-la-Neuve, Belgium; 5 Institute for Tropical Medicine, Antwerp, Belgium; 6 Department of Veterinary Tropical Diseases, University of Pretoria, Pretoria, South Africa; Centro de Pesquisas René Rachou, BRAZIL

## Abstract

*Rhipicephalus microplus* is widely distributed in tropical and subtropical areas of the world where livestock is a principal activity with great veterinary and economic importance. In Ecuador, this hematophagous ectoparasite has been observed between 0 and 2600 masl. One of the main tick control measures is the use of acaricides, which have been indiscriminately used worldwide and in Ecuador. In this country, no studies on acaricide resistance in *Rhipicephalus microplus* have been published. The current study aims to characterise the level of resistance of *R*. *microplus* against three main acaricides commonly used in Ecuador i.e. amitraz, alpha-cypermethrin and ivermectin to determine the level and pattern of dose-responses for *R*. *microplus* in 12 field populations (farms). The level of acaricide resistance was evaluated using three different bioassays: adult immersion test (AIT), larval package test (LPT) and larval immersion test (LIT), as recommended by the FAO. The predictive dose-responses were analysed by binomial logistics regression of the larval survival rate (resistance). In general, we found resistance of 67% for amitraz; 50% for alpha-cypermethrin and from 25 to 42% for ivermectin in the twelve field populations analysed. Resistance levels were studied in larval and adult bioassays, respectively, which were slightly modified for this study. For larval bioassays based on corrected mortality i.e. high (above 51%), medium (21–50%) and low (11–20%) resistance, percentages less than 10% were considered as susceptible. For the adult test, two resistance levels were used i.e. high (more than 76%) and medium (51 to 75%) resistance. Percentages lower than 50% were considered as susceptible. In this context, for larval bioassays, amitraz showed 21%, 38% and 8% for high, medium and low resistance, respectively. Alpha-cypermethrin presented 8%, 4 and 38% for high, medium and low resistance, respectively. Ivermectin presented 8%, 25% and 8% for high, medium and low resistance, respectively. For adult tests with amitraz 50% and 17% of the field populations showed average and high resistance, with evidences of average resistance to alpha-cypermethrin in 50% of the samples and average resistance against ivermectin in 25% of the farms. No statistical difference amongst the three bioassays was found and acaricide resistance was confirmed by logistic regression analysis; hence resistance (dose-responses) in each field populations differed, depending on the choice of the acaricide, frequent usage, frequency of treatment and farm management. The effective estimated dose needed to eliminate 99% of ticks is higher than dose recommended by the manufacturer. In conclusion, amitraz showed the highest resistance followed by ivermectin and alpha-cypermethrin and reveals differences on resistance in each individual field population. This information is important in order to establish the monitoring of resistance on each farm individually, contributing to the rational use of acaricides included in an integrated control program for *R*. *microplus*.

## Introduction

In Ecuador, livestock farming is one of the main economic activities with 75% of Ecuadorian livestock farms located in tropical and subtropical coastal areas and in the Amazon region. These farms are infested or at risk to be infested by ticks [1], and acaricidal treatment are part of their control programs [2]. *Rhipicephalus microplus* is the main cattle tick in Ecuador, and is distributed between 0 and 2600 meters above sea level (masl) [3,4]. *Amblyomma* spp. and *Ixodes* spp. are present but are less important than *Rhipicephalus microplus*.

Since the second half of the 1990s acaricide resistance has been observed and since then it is very common against all commercially available products, resulting in serious economic losses [5,6]. Acaricide resistance is caused by several intrinsic and operational factors [7,8]. Intrinsic factors are related to the biology, ecology, genetics, and the mutation rate of ticks, while operational factors are management-related [8,9].

The presence of resistant populations has been described worldwide and associated with significant livestock and public health problems. In Brazil, Mexico, and Colombia, several studies have been undertaken, showing the resistance and/or vulnerability to organ-phosphorated and organ-chlorinated compounds, pyrethroids (deltamethrin, cypermethrin, flumethrin, alpha-cypermethrin, and lambda-cyhalothrin), methyl carbamate and recently ivermectin [10–12]. According to observations of the "National Survey of Brucellosis, Bovine Tuberculosis and Ticks" carried out by the Public Health and Zoonosis Research Institute (unpublished data), amitraz, ivermectin and a recent alternative, alpha-cypermethrin, [2] are the acaricides extensively used in Ecuador in about 42%, 39%, 24% of farms, respectively. Additionally, farmers reported in a survey that amitraz, with different brand names, is commonly used in the study area in various doses, concentrations, intervals between treatments and application methods (unpublished data). However, no study of resistance had so far been formally carried out in Ecuador. The north-western region of Ecuador has ideal conditions to assess acaricide resistance and the dose-response on field populations of ticks. It is a tropical region with a high density of livestock that fosters the development of *R*. *microplus*. To fulfil the objectives of this study, chemical bioassays i.e. adult immersion test, larval immersion test, and larval package test, are used as reported in the literature [13–15] and suggested by the World Health Organization (WHO). The dose-responses were analysed by binomial logistic regression of the larval survival rate (resistance) as described in similar studies [16–18]. Detection of resistance in field populations is essential to establish integrated control programs to delay its development and to ensure a sustainable use of acaricides [14,19]. Future studies will then allow evaluating the effectiveness of acaricides, by determining the effect of their application on the selection of individual tick populations, and by the study of resistance genes and the evolutionary potential of ticks in the area. To our knowledge, this study is the first report on the evaluation of the current situation of resistance levels of ivermectin, amitraz and alpha-cypermethrin in Ecuador. The detection of resistance in each farm, together with its history of acaricide use, can give valuable information to manage the control of ticks and evaluate acaricide resistance in the farm [19]. Lastly, from the economic and social perspective, this study will facilitate the proposal of a rational, adequate, and effective use of chemical control associated with appropriate integrated control strategies involving farmers, pharmaceutical companies, and veterinarians.

## Materials and methods

### Study areas and sampling

**Fig 1:**
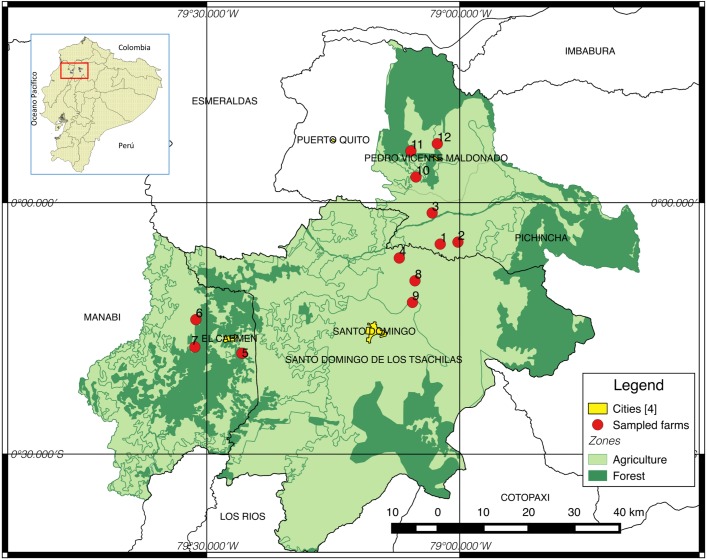
Study area. Twelve livestock dairy farms distributed in four areas of the coastal area of Ecuador were surveyed. Farm range temperatures between 15 and 35°C and an altitude from 500 to 1,500 masl. Source: MAGAP (2017): http://geoportal.sigtierras.gob.ec:8080/GeoserverViewer/.

The study was undertaken on 12 livestock dairy where a field populations were collected from each. The farms are distributed in four areas: three in San Miguel de los Bancos county, three in Pedro Vicente Maldonado county, three in Santo Domingo de los Tsáchilas county and three in El Carmen county. The farms were located in the coastal area of Ecuador with temperatures ranging from 15 to 35°C and an altitude from 200 to 1,500 masl [20] (Fig 1). Ticks were collected on each farm from October to December 2015, following the methods described by Junte in 2008 [21]. Farms were selected based on their history of use of acaricides i.e. different acaricide types, concentration, combinations and application frequencies that were different in each farm. The number of animals with ticks and livestock management systems were also considered. In addition, none or few technical criteria were reported by farmers; hence the conditions to evaluate acaricides resistance was ideal for this study. Each farm was surveyed and geographically referenced with a Garmin GPSmap64®. For this study, no specific permissions were required because ticks a serious problem for farmers and any endangered or protected species were involved.

### Field populations samples

After receiving the authorization of the farmer, ticks were collected directly from animals, by moving the hand slowly over the animals and catching those that were spontaneously released, or softly removed by traction, without breaking the hypostoma, an important structure for the identification and survival of the tick [22,23]. From each farm, 250 live engorged female ticks, over 5 mm long, were collected. Maximum 25 engorged females were placed in a labelled plastic jar, with punctured lids for air circulation and with slightly-moistened cotton inside [14,22,24]. After this, samples were transported, within two days at most, to the Public Health and Zoonosis Research Institute, where they were placed under controlled conditions until different bioassays were carried out [14].

### Chemicals

As different brands, presentations, combinations and concentrations of the commercial products exist, the active ingredients of amitraz, ivermectin, and alpha-cypermethrin were obtained from the pharmaceutical companies and diluted according to their recommendations. i.e. amitraz and alpha-cypermethrin were diluted in 10 ml xylene (w/v), ivermectin in glycerine (w/v) and stored as stock dilution (100x each). Protected against direct sunlight or humidity, dilutions were stable for around 6 months. Concentrations used for predictive dose-response were: alpha-cypermethrin at 0.002%, 0.02%, and 0.5%; amitraz at 0.002%, 0.1%, and 0.25% and ivermectin at 0.01%, 0.1%, and 0.5% for the minimum, medium or discriminatory and maximum doses. Stock solutions were diluted in distilled water 10 minutes before use. Concentrations were changed and/or adapted based on the FAO in 2004 [14] and Rivera recommendations [22]. The discriminatory dose used in this study is the dose based on which the product is sold in Ecuador.

### Bioassays

Ticks were identified using dichotomous keys proposed by Barros-Battesti et al.,[25] Cooley [26], Keirans and Durden [27], Martins et al. [28] and Vargas [29]. For the handling of active ingredients, all biosecurity measures were applied for class 3 toxic substances [14,22]. Once in the lab, ticks were carefully cleaned to prevent secondary contamination and placed in hatcheries at 27°C, and 80% relative humidity, until 3 bioassays were carried out [14] i.e. around two days for the adult test and three weeks for the larval tests. Tests were carried out to determine if acaricides affected the hatching of eggs. Two tests were carried out to determine the mortality rate of larvae.

Adult immersion test (AIT): The protocol was described by Drummond et al. [13], and adapted by the FAO in 2004 [14]. In total, 10 homogeneous groups were formed for 10 engorged females larger than 5 mm coming from the same farm. Each group was submerged for three minutes in different concentrations of each of the three acaricides. A control group submerged in distilled water was included. After this, specimens were dried for thirty minutes on absorbent paper, dorsally immobilized on adhesive tape strips, and placed in Petri dishes. Petri dishes were placed in hatching chambers at 27°C and 85% relative humidity in preparation for oviposition [30]. The mortality rate and oviposition were registered on the seventh day of the bioassay, egg mass was then collected and individually weighed. They were placed in 2-ml cryovial tubes, until hatching.

Larval immersion test (LIT): this bioassay was described and modified by Mekonnen et al. in 2002 and 2003 [15,31]. In total, around 100 larvae were used per bioassay and per concentration, ranging from 14 to 21 days after hatching, after the complete development and the start of their infective stage according to Gallardo in 1999 [32] and Anderson and Magnarelli in 2008 [33]. Larvae were carefully placed, using brushes, in different concentrations of acaricides, for 10 minutes. Additionally, a control group was submerged in distilled water [10]. Then they were dried on absorbent paper for 30 minutes, and subsequently packaged in individual envelopes, sealed and placed in the hatchery at 27°C and 85% relative humidity. The mortality rate was read 72 hours later [10,21].

Larval package test (LPT) (described by FAO in 2004 [14]): as for the LIT bioassay, around 100 larvae, from 14 to 21 days after hatching, were exposed to filter papers that had been previously imbued with different concentrations of ivermectin and alpha-cypermethrin. For amitraz, a nylon base was used. For alpha-cypermethrin and ivermectin, packages were opened after 24 hours and for amitraz after 48 hours, to count live and total number of larvae [14,34].

### Data analysis

Resistance of adults, the corrected mortality rate for LIT and LPT were calculated as described by FAO in 2004 [14] and Junte in 2008 [21]. To calculate the presence and degree of resistance for each farm, results were interpreted for percentages equal or greater than 10% of larval survival (for LPT and LIT tests) and equal or higher than 50% of resistance for adult ticks (AIT), according to Junte in 2008 [21] slightly modified (Table 1).

**Table 1:** Interpretation of acaricide resistance levels in ticks collected in farms in Ecuador.

	Larvae (Survival %)^d^	Adults (Resistance %)
Category	LPT^a^ and LIT^b^	AIT^c^
Susceptible	<10%	<50%
Low Resistance	11% - 20%	-
Medium Resistance	21% - 50%	51% - 75%
High Resistance	>51%	>76%

^a^Larval Package Test.

^b^Larval Immersion Test.

^c^Adult Immersion Tests.

^d^100 minus the corrected death rate.

The corrected values were calculated as follows

Adult immersion test:

Reproductive estimate (ER)

$$ER=\frac{Total\;weight\;of\;eggs\;(g)*\;estimated\;hatching\;(\%)*\;20,000\;(\#\;of\;eggs)\;}{number\;of\;females}$$ (1)

Calculation of the correction of ER compared to the control group.

$$(\%)Control=\frac{(ER\;control\;ticks-ER\;treated\;ticks)*100}{ER\;treated\;ticks}$$ (2)

Resistancepercentage(%)=100−control(%) (3)

For larvae:

$$Corrected\;death\;rate\;for\;larvae\;tests\;(LPT\;and\;LIT)=\frac{(mortality\;rate\;of\;the\;test-mortality\;rate\;of\;control\;group)\;*\;100}{100-\%\;death\;rate\;of\;the\;control\;group}$$ (4)

### Statistical analysis

A multi-variance analysis was carried out to determine possible significant differences between LIT and LPT tests [35,36]. The Predictive dose-responses were analysed by binomial logistics regression of the larval survival rate (resistance) using the Statistic "R" free software version 3.2.3 and the “dcr” statistical package (S1 and S2 R Analysis) [37,16,17].

## Results

### Bioassays

The adult immersion test showed resistance values of 67%, 50%, and 25% in *R*. *microplus* for Amitraz, alpha-cypermethrin and ivermectin, respectively. Resistance in field populations by LIT and LPT bioassays were 67%, 50% and 42%, for amitraz, alpha-cypermethrin and ivermectin, respectively (Table 2).

**Table 2:** Results of bioassays of acaricides: ticks resistance by acaricides, by bioassays and by farms, in Ecuador.

Acaricides/Bioassay									
Farm	Amitraz			Alpha-cypermethrin			Ivermectin		
	AIT^a^	LIT^b^	LPT^c^	AIT	LIT	LPT	AIT	LIT	LPT
1	x	x	x		x	x	x	x	x
2					x	x	x	x	x
3	x	x	x	x				x	x
4	x	x	x	x	x	x			
5	x	x	x	x	x	x			
6	x	x	x	x	x	x			
7	x	x	x						
8									
9	x	x	x				x	x	x
10				x	x	x			
11	x	x	x						
12				x				x	x
Total	8	8	8	6	6	6	3	5	5
%^d^	67	67	67	50	50	50	25	42	42

^a^Larval Package Test.

^b^Larval Immersion Test.

^c^Adult Immersion Tests.

^d^Percentages of resistance per each acaricide and bioassay in the study.

Table 2 shows the presence or absence of resistance reported by field populations, for different chemical products and types of bioassays. All field populations, except one, showed resistance to one or more chemical products. It is important to mention that one field population showed resistance against the three acaricides, and six against two i.e. three field populations against amitraz and alpha-cypermethrin and two to alpha-cypermethrin and ivermectin and one to amitraz and ivermectin. In four field populations there was resistance to only one product i.e. two to amitraz, one to alpha-cypermethrin and one to ivermectin.

### Resistance toxicological analysis

**Fig 2:**
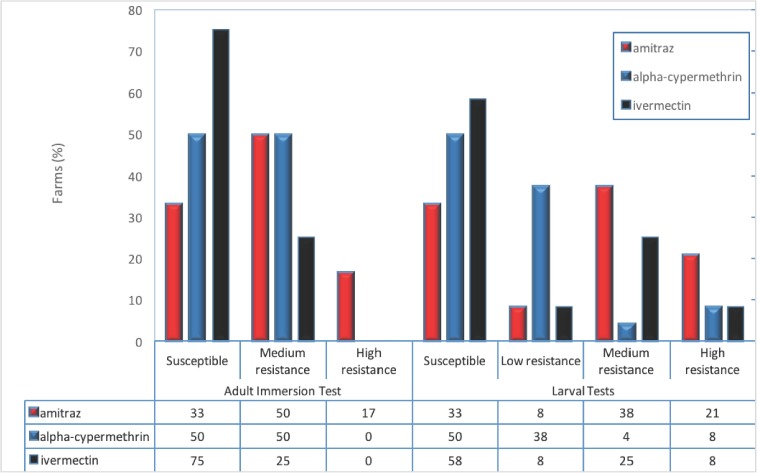
Resistance Levels per each acaricide and bioassay. Resistance Levels for Adult Immersion Test were divided in three categories i.e. Susceptible (<50%), Medium resistance (51 to 75%) and High resistance (>76%); for Larval Test, four categories were identified i.e. high (>51%), medium (21–50%) and low (11–20%) resistance and Susceptible (<10%). Larval Package Test and Larval Immersion Test were applied to assess resistance levels on larvae; no significant difference was found between bioassays (p > 0.05). For this reason, both bioassays were combined in one figure.

No evidence of resistance was found with adult immersion test, (Fig 2) in 33.3% of the field populations but average and high resistance in *R*. *microplus* was observed in 50% and 16.7% of the farms. There were no evidences of resistance to alpha-cypermethrin in 50% of the samples and 50% showed an average resistance. In nine field populations resistance against ivermectin (75%) was not found, and three showed an average resistance (25%). For adults, resistance was greater to amitraz, followed by alpha-cypermethrin and to ivermectin (respectively in 8, 6 and 3 field populations).

When two bioassays were applied to larvae, no significant difference was found for treatments (p > 0.05), in agreement with Junte, (2008). For larvae, susceptibility to amitraz, alpha-cypermethrin and ivermectin, was found in four (33%), six (50%), and seven (58.3%) field populations, respectively. The general resistance percentages for the three acaricides were 66.6% (eight populations), 50% (six populations) and 41.7% (five populations), respectively for amitraz, alpha-cypermethrin and ivermectin (see Fig 2).

### Dose-response

**Fig 3:**
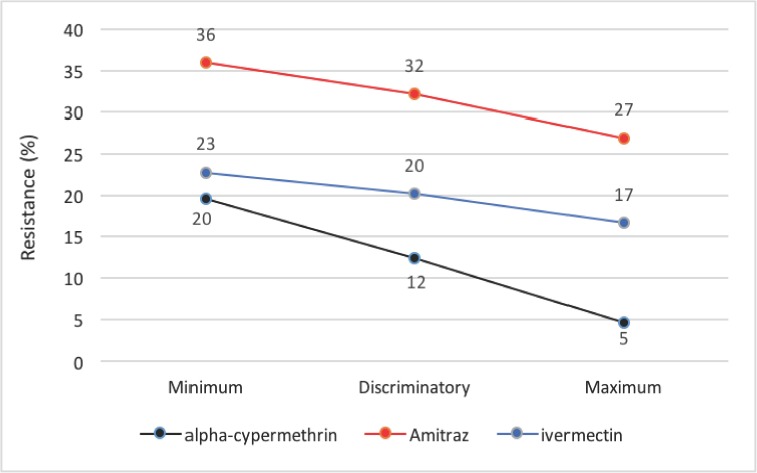
Dose-response to amitraz, alpha-cypermethrin and ivermectin. In general terms, acaricide resistance is important in the study area. Except for the alpha-cypermethrin maximum dose, none of the doses i.e. minimum, discriminatory and maximum doses, killed the 100% of ticks. All doses showed resistance.

Results from minimun, discriminatory and maximum doses are given in Fig 3. None of the doses killed the 100% of ticks even at maximum dosage which is supposed to be toxic. In our study, in spite of using hypertoxic doses, survival rates were found in 27%, 5%, and 17% of ticks using respectively amitraz, alpha-cypermethrin and ivermectin. This is most likely due to the resistance of *R*. *microplus* to acaricides as demonstrated in this study.

**Fig 4:**
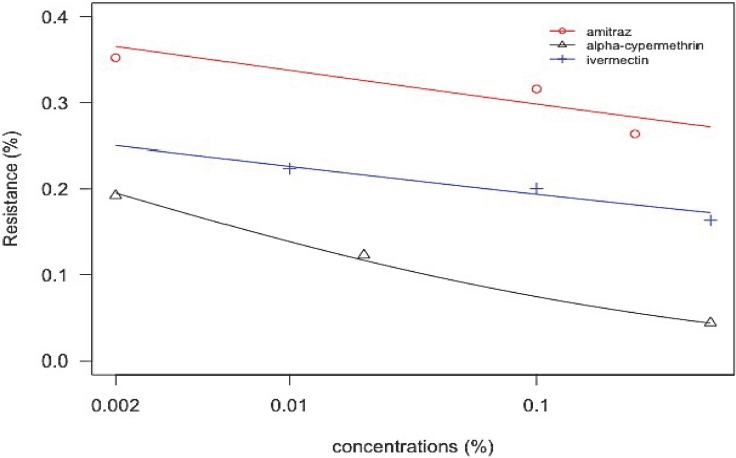
Predicted Dose-response to amitraz, alpha-cypermethrin and ivermectin. Predicted Dose-response analysis was analysed by binomial logistics regression of the larval survival rate (resistance) and based on the minimum, discriminatory and maximum doses as recommended by FAO. Probit analysis corroborate the findings found in our study.

The predicted dose-response (Fig 4 and S1 R Analysis) was analysed using a logistic regression on field populations for both larval bioassays jointly.

**Fig 5:**
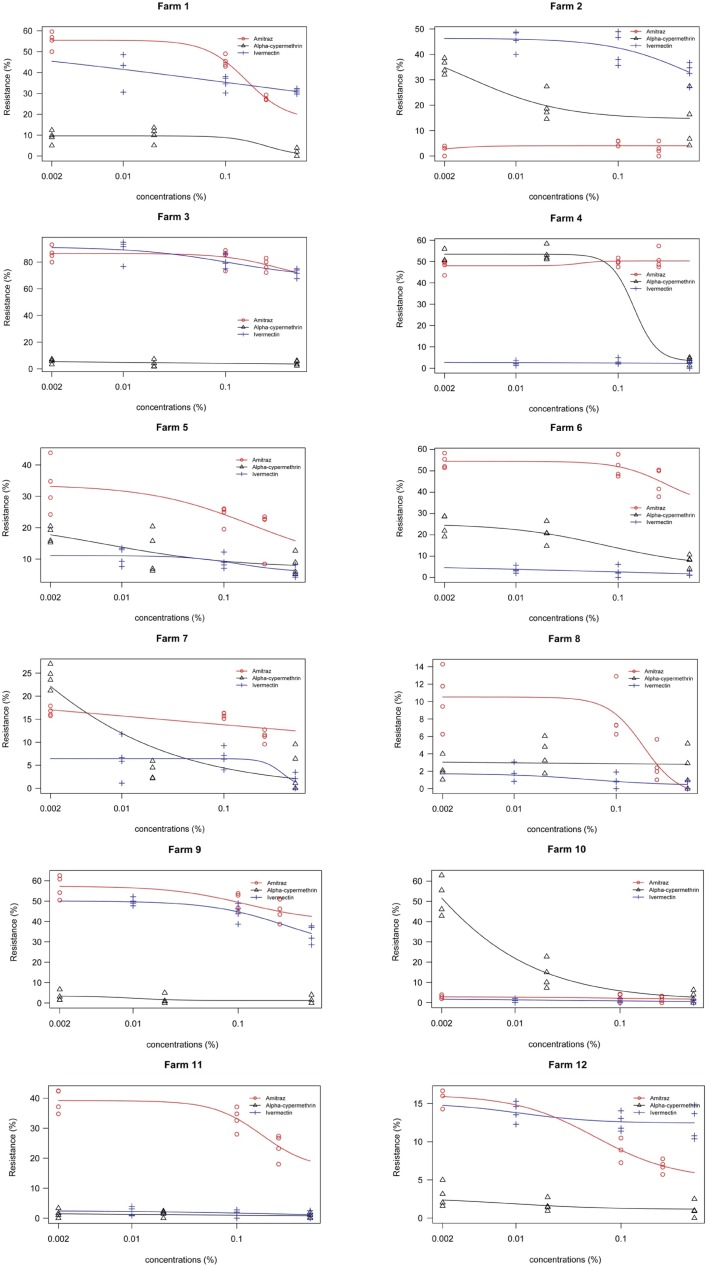
Predictive dose-response confirm the results in each bioassay per farm. Analysis were based on three different concentrations per acaricide i.e. alpha-cypermethrin at 0.002%, 0.02%, and 0.5%; amitraz at 0.002%, 0.1%, and 0.25% and ivermectin at 0.01%, 0.1%, and 0.5% for the minimum, medium or discriminatory and maximum doses, respectively. All farms except farm 8 showed resistance levels (more than 10%) according to use frequency of each acaricide.

In general, results for each field population confirmed the trends obtained for each bioassay (LPT and LIT). In 92% of cases (Fig 5 and S2 R Analysis), the increase in death rates corresponds with the dose concentration.

Likewise, the predicted dose-response analysis allowed us to calculate the Estimated Effective Dose (EED) for each active ingredient that would kill 99% of ticks i.e. the "discriminatory dose", and this appeared to be greater than 10 times the normal concentration of commercially-sold acaricides, a concentration too toxic for the hosts and for the environment [38].

## Discussion

All bioassays performed in this study demonstrated elevated resistance in *R*. *microplus* to amitraz, alpha-cypermethrin and ivermectin. According to these protocols as defined by the FAO (2004), the resistance percentage was based on the calculation of the percentage of eggs produced per female and the percentage of hatching eggs or surviving larvae (Table 2). Resistance was found for each of these criteria. As expected, resistance values are higher for amitraz than for other compounds. This relates to the continuous and massive use of amitraz in the 1990’s [2]. Amitraz and alpha-cypermethrin are the recommended acaricides to control ticks. In Ecuador, ivermectin is used to control external and internal parasites but its use in production animals is forbidden, because of its residual effect. This is probably the reason why resistance to this acaricide is lower than amitraz but higher than alpha-cypermethrin. In this survey, one population did not show resistance to any of the products tested. This likely relates to the farm management system relying on chemical products different from those tested in this study.

The situation observed in this study is consistent with observations in other Latin American countries. In Colombia, Araque et al., (2014) [39] reported resistance to amitraz in 97% of 71 farms. In Venezuela, Coronado and Mujica, (1997) [40] reported resistance to synthetic pyrethroids. In Cuba, Valle et al., (2004) [41] reported resistance to cypermethrin, amitraz, and ivermectin, although without specific data. In Mexico, resistance to amitraz has also been described [42]. In Uruguay, resistance to ivermectin in *R*. *microplus* has been reported [43], while in Brazil, Andreotti et al., (2011) [44] using the adult immersion test found resistance to alpha-cypermethrin, cypermethrin, and amitraz.

Although AIT calculates mortality/survival differently from larval test, resistance levels, observed in *R*. *microplus* collected in this study, showed similar results except for ivermectin; for AIT resistance was observed in three populations, the larval test in five populations. In general, resistance appeared to be higher for amitraz, followed by alpha-cypermethrin, and lastly to ivermectin.

The situation observed in the field populations with the highest levels of resistance suggests that the resistance may increase everywhere and worsen in the near future if adequate control measures are not taken. The Ecuadorian Health Authority details its recommendations in a technical proposal for management in each individual farm, complemented by adequate training on the use and management of acaricides i.e. an integrated control program.

A major concern raised by our results is that even doses much higher than the discriminatory doses could not cause a 99.9% mortality as defined by Constant and Roush, (1990). The resistance values of ticks to different acaricides are based on results with doses recommended by the manufacturers as indicated as the "discriminatory dose" or the "stock dose," which theoretically corresponds to DL_99_. This means that it should eliminate 99.9% of the total tick population [45]. In this study, the minimum and maximum doses, recommended by FAO, were included to relate and corroborate results obtained by the discriminatory dose (see Fig 5 and S2 R Analysis). Additionally, it is expected that acaricides used at low concentrations will allow a greater survival rate, while for maximum concentrations, the death rate should be 100%. In our study, the expected results were not found. This corroborates observations of other authors [11,39,46,47], showing that acaricides that have been frequently used and for long periods are causing a greater resistance, compared to those that have been recently introduced in the market, whose frequency of use is not significant yet.

Results were heterogeneous among field populations. On each farm, ticks reacted in a different manner probably due to the individual behaviour based on the management of pastures, nutrition and the use of acaricides, which might favour the selection pressure on the population. It is common practice that, when acaricide resistance increases, and when no new products are available, pharmaceutical companies have been forced to modify the recommended concentrations which consequently makes them more toxic to the hosts, and the environment. E.g. in the 1980’s, the concentration of ivermectin used was 33 times less than the one currently used (0.003% vs. 0.1%; [48]). This pattern of resistance has not been reported previously probably because other resistance studies concentrated on regional situations rather than individual farm problems (Fig 5).

The analysis of the dose-response [37,16,17] allows us to predict the outcome of the use of acaricides in this study. Jonsson et al., [49] and Mendes et al. [19], found it unlikely that AIT can be an effective screening test in the field, being only indicative. For this reason and to obtain a better explanation, the logistics regression analysis was used for every field population and jointly, for larval bioassays. In some cases, it was observed that acaricides affected the mortality rate of ticks, although the effect was slightly unexpected. Maybe this is due to problems in acaricide handling for each bioassay or when lab steps were performed.

In this study, resistance in *R*. *microplus* against amitraz was present in 67% of the farms under study, for adult immersion and larval package tests, respectively, and 75% for larval immersion. Resistance to alpha-cypermethrin was detected in 50% of the farms, by adult immersion and larval package tests and in 42% of the farms by larval immersion. Finally, for ivermectin, resistance was found in 25% of farms by adult immersion tests and in 42% by larval immersion tests and in 50% by larval package tests. The data obtained in this study is alarming, considering that acaricide resistance is likely widespread over the tropical and subtropical areas of Ecuador, with farms showing a multi-resistance profile (Fig 5), encompassing all of the three acaricides available on the market.

This study aimed to provide information to improve control measures which should be implemented, using products whose effectiveness has been proven accross farm, combined with alternatives such as pasture management, field rotation, integrated pest management strategies such as vaccines, biological control agents, and others in order to decrease acaricide dependency and reduce genetic changes caused by selection pressure [50].

## Supporting information

S1 R Analysis Predictive dose-response per drug. Analysis were based on three different concentrations per acaricide i.e. alpha-cypermethrin at 0.002%, 0.02%, and 0.5%; amitraz at 0.002%, 0.1%, and 0.25% and ivermectin at 0.01%, 0.1%, and 0.5% for the minimum, medium or discriminatory and maximum doses, respectively. In general, the three acaricide products show resistance in all levels.
(PDF)

S2 R Analysis Predictive dose-response per farm. Analysis were based on three different concentrations per acaricide i.e. alpha-cypermethrin at 0.002%, 0.02%, and 0.5%; amitraz at 0.002%, 0.1%, and 0.25% and ivermectin at 0.01%, 0.1%, and 0.5% for the minimum, medium or discriminatory and maximum doses, respectively. All farms except farm 8 showed resistance levels (more than 10%) according to use frequency of each acaricide
(PDF)
