# Supplementary material for: The current status of resistance to alpha-cypermethrin, ivermectin, and amitraz of the cattle tick (Rhipicephalus microplus) in Ecuador
Source: PLoS One. 2017 Apr 7;12(4):e0174652. doi: 10.1371/journal.pone.0174652 (PMC5384665; doi:10.1371/journal.pone.0174652)
Supplement: S1 R Analysis — Analysis were based on three different concentrations per acaricide i.e. alpha-cypermethrin at 0.002%, 0.02%, and 0.5%; amitraz at 0.002%, 0.1%, and 0.25% and ivermectin at 0.01%, 0.1%, and 0.5% for the minimum, medium or discriminatory and maximum doses, respectively. In general, the three acaricide products show resistance in all levels. (PDF) [file pone.0174652.s001.pdf]

Predicted_dose_response_per_drug.R

nuinuce

Sun Jan 22 19:59:36 2017

```r
##Analysis were based on three different concentrations per acaricide
#i.e. alpha-cypermethrin at 0.002%, 0.02%, and 0.5%; amitraz at 0.002%, 0.1%, and 0.25%
#and ivermectin at 0.01%, 0.1%, and 0.5% for the minimum, medium or discriminatory
#and maximum doses, respectively. In general, the three acaricide products show
#resistance in all levels

library(drc)
```

```
## Loading required package: MASS
##
## 'drc' has been loaded.
##
## Please cite R and 'drc' if used for a publication,
## for references type 'citation()' and 'citation('drc')'.
##
##
## Attaching package: 'drc'
##
## The following objects are masked from 'package:stats':
##
##     gaussian, getInitial
```

```r
Predicdrug<-read.csv("totalresist.csv",header = T,sep = ";", dec = ",")

Predicdrug
```

```
##                    Localidad  finca              droga  conc totalX
## 1                 Los Bancos  Fin 1            amitraz 0.002 100.50
## 2                 Los Bancos  Fin 1            amitraz 0.100  98.75
## 3                 Los Bancos  Fin 1            amitraz 0.250 100.25
## 4                 Los Bancos  Fin 1 alpha-cypermethrin 0.002 101.25
## 5                 Los Bancos  Fin 1 alpha-cypermethrin 0.020 100.50
## 6                 Los Bancos  Fin 1 alpha-cypermethrin 0.500 107.50
## 7                 Los Bancos  Fin 1         ivermectin 0.010 101.00
## 8                 Los Bancos  Fin 1         ivermectin 0.100  88.25
## 9                 Los Bancos  Fin 1         ivermectin 0.500  89.50
## 10                Los Bancos  Fin 2            amitraz 0.002  89.50
## 11                Los Bancos  Fin 2            amitraz 0.100 101.50
## 12                Los Bancos  Fin 2            amitraz 0.250 101.00
## 13                Los Bancos  Fin 2 alpha-cypermethrin 0.002 110.50
## 14                Los Bancos  Fin 2 alpha-cypermethrin 0.020 105.00
## 15                Los Bancos  Fin 2 alpha-cypermethrin 0.500 106.25
## 16                Los Bancos  Fin 2         ivermectin 0.010 100.00
## 17                Los Bancos  Fin 2         ivermectin 0.100 101.75
## 18                Los Bancos  Fin 2         ivermectin 0.500  80.50
## 19                Los Bancos  Fin 3            amitraz 0.002 110.75
```

```
## 20         Los Bancos  Fin 3          amitraz 0.100 119.50
## 21         Los Bancos  Fin 3          amitraz 0.250 120.00
## 22         Los Bancos  Fin 3 alpha-cypermethrin 0.002 109.25
## 23         Los Bancos  Fin 3 alpha-cypermethrin 0.020 123.75
## 24         Los Bancos  Fin 3 alpha-cypermethrin 0.500 102.25
## 25         Los Bancos  Fin 3         ivermectin 0.010  99.25
## 26         Los Bancos  Fin 3         ivermectin 0.100 108.75
## 27         Los Bancos  Fin 3         ivermectin 0.500 114.25
## 28      Santo Domingo  Fin 4          amitraz 0.002  94.75
## 29      Santo Domingo  Fin 4          amitraz 0.100  95.25
## 30      Santo Domingo  Fin 4          amitraz 0.250  97.25
## 31      Santo Domingo  Fin 4 alpha-cypermethrin 0.002  92.50
## 32      Santo Domingo  Fin 4 alpha-cypermethrin 0.020  93.75
## 33      Santo Domingo  Fin 4 alpha-cypermethrin 0.500  90.25
## 34      Santo Domingo  Fin 4         ivermectin 0.010  97.50
## 35      Santo Domingo  Fin 4         ivermectin 0.100  98.75
## 36      Santo Domingo  Fin 4         ivermectin 0.500  93.25
## 37          El Carmen  Fin 5          amitraz 0.002  95.75
## 38          El Carmen  Fin 5          amitraz 0.100  95.00
## 39          El Carmen  Fin 5          amitraz 0.250  94.50
## 40          El Carmen  Fin 5 alpha-cypermethrin 0.002  94.50
## 41          El Carmen  Fin 5 alpha-cypermethrin 0.020 101.00
## 42          El Carmen  Fin 5 alpha-cypermethrin 0.500  93.75
## 43          El Carmen  Fin 5         ivermectin 0.010  97.50
## 44          El Carmen  Fin 5         ivermectin 0.100  95.25
## 45          El Carmen  Fin 5         ivermectin 0.500  98.25
## 46          El Carmen  Fin 6          amitraz 0.002 106.00
## 47          El Carmen  Fin 6          amitraz 0.100 118.25
## 48          El Carmen  Fin 6          amitraz 0.250 108.00
## 49          El Carmen  Fin 6 alpha-cypermethrin 0.002 114.25
## 50          El Carmen  Fin 6 alpha-cypermethrin 0.020 115.75
## 51          El Carmen  Fin 6 alpha-cypermethrin 0.500 106.75
## 52          El Carmen  Fin 6         ivermectin 0.010  96.50
## 53          El Carmen  Fin 6         ivermectin 0.100 101.25
## 54          El Carmen  Fin 6         ivermectin 0.500  94.75
## 55          El Carmen  Fin 7          amitraz 0.002  96.25
## 56          El Carmen  Fin 7          amitraz 0.100  99.00
## 57          El Carmen  Fin 7          amitraz 0.250  92.50
## 58          El Carmen  Fin 7 alpha-cypermethrin 0.002 121.75
## 59          El Carmen  Fin 7 alpha-cypermethrin 0.020  92.50
## 60          El Carmen  Fin 7 alpha-cypermethrin 0.500  93.00
## 61          El Carmen  Fin 7         ivermectin 0.010  83.25
## 62          El Carmen  Fin 7         ivermectin 0.100 100.25
## 63          El Carmen  Fin 7         ivermectin 0.500  95.00
## 64      Santo Domingo  Fin 8          amitraz 0.002 102.25
## 65      Santo Domingo  Fin 8          amitraz 0.100 114.50
## 66      Santo Domingo  Fin 8          amitraz 0.250 107.75
## 67      Santo Domingo  Fin 8 alpha-cypermethrin 0.002 106.25
## 68      Santo Domingo  Fin 8 alpha-cypermethrin 0.020 120.00
## 69      Santo Domingo  Fin 8 alpha-cypermethrin 0.500 106.75
## 70      Santo Domingo  Fin 8         ivermectin 0.010 112.75
## 71      Santo Domingo  Fin 8         ivermectin 0.100 118.75
## 72      Santo Domingo  Fin 8         ivermectin 0.500 106.75
## 73      Santo Domingo  Fin 9          amitraz 0.002 117.50
```

```
## 74             Santo Domingo  Fin 9            amitraz 0.100 106.75
## 75             Santo Domingo  Fin 9            amitraz 0.250 110.75
## 76             Santo Domingo  Fin 9 alpha-cypermethrin 0.002 126.50
## 77             Santo Domingo  Fin 9 alpha-cypermethrin 0.020 107.50
## 78             Santo Domingo  Fin 9 alpha-cypermethrin 0.500 118.25
## 79             Santo Domingo  Fin 9         ivermectin 0.010 117.25
## 80             Santo Domingo  Fin 9         ivermectin 0.100 116.75
## 81             Santo Domingo  Fin 9         ivermectin 0.500 114.50
## 82  Pedro Vicente Maldonado Fin 10            amitraz 0.002 102.25
## 83  Pedro Vicente Maldonado Fin 10            amitraz 0.100 107.50
## 84  Pedro Vicente Maldonado Fin 10            amitraz 0.250 117.25
## 85  Pedro Vicente Maldonado Fin 10 alpha-cypermethrin 0.002 108.75
## 86  Pedro Vicente Maldonado Fin 10 alpha-cypermethrin 0.020 112.00
## 87  Pedro Vicente Maldonado Fin 10 alpha-cypermethrin 0.500 105.75
## 88  Pedro Vicente Maldonado Fin 10         ivermectin 0.010 120.25
## 89  Pedro Vicente Maldonado Fin 10         ivermectin 0.100 109.25
## 90  Pedro Vicente Maldonado Fin 10         ivermectin 0.500 106.50
## 91  Pedro Vicente Maldonado Fin 11            amitraz 0.002 111.00
## 92  Pedro Vicente Maldonado Fin 11            amitraz 0.100 106.25
## 93  Pedro Vicente Maldonado Fin 11            amitraz 0.250 110.50
## 94  Pedro Vicente Maldonado Fin 11 alpha-cypermethrin 0.002  78.00
## 95  Pedro Vicente Maldonado Fin 11 alpha-cypermethrin 0.020 109.00
## 96  Pedro Vicente Maldonado Fin 11 alpha-cypermethrin 0.500  99.50
## 97  Pedro Vicente Maldonado Fin 11         ivermectin 0.010 113.25
## 98  Pedro Vicente Maldonado Fin 11         ivermectin 0.100  98.00
## 99  Pedro Vicente Maldonado Fin 11         ivermectin 0.500 107.00
## 100 Pedro Vicente Maldonado Fin 12            amitraz 0.002 111.50
## 101 Pedro Vicente Maldonado Fin 12            amitraz 0.100 118.00
## 102 Pedro Vicente Maldonado Fin 12            amitraz 0.250 124.50
## 103 Pedro Vicente Maldonado Fin 12 alpha-cypermethrin 0.002  84.75
## 104 Pedro Vicente Maldonado Fin 12 alpha-cypermethrin 0.020 122.25
## 105 Pedro Vicente Maldonado Fin 12 alpha-cypermethrin 0.500 107.00
## 106 Pedro Vicente Maldonado Fin 12         ivermectin 0.010  99.25
## 107 Pedro Vicente Maldonado Fin 12         ivermectin 0.100 125.75
## 108 Pedro Vicente Maldonado Fin 12         ivermectin 0.500 109.50
##     muertosX vivosX
## 1      44.75  55.75
## 2      54.00  44.75
## 3      72.50  27.75
## 4      92.00   9.25
## 5      90.25  10.25
## 6     106.00   1.50
## 7      59.00  42.00
## 8      57.25  31.00
## 9      61.75  27.75
## 10     87.00   2.50
## 11     96.00   5.50
## 12     98.25   2.75
## 13     72.00  38.50
## 14     84.25  20.75
## 15     90.50  15.75
## 16     54.00  46.00
## 17     58.50  43.25
## 18     54.00  26.50
```

```
## 19     15.00  95.75
## 20     19.50 100.00
## 21     26.25  93.75
## 22    103.00   6.25
## 23    118.75   5.00
## 24     98.25   4.00
## 25     10.75  88.50
## 26     21.50  87.25
## 27     31.75  82.50
## 28     49.25  45.50
## 29     48.00  47.25
## 30     47.75  49.50
## 31     43.25  49.25
## 32     43.50  50.25
## 33     87.00   3.25
## 34     95.25   2.25
## 35     95.75   3.00
## 36     91.50   1.75
## 37     64.00  31.75
## 38     72.00  23.00
## 39     76.25  18.25
## 40     77.75  16.75
## 41     88.75  12.25
## 42     86.25   7.50
## 43     86.75  10.75
## 44     86.50   8.75
## 45     92.00   6.25
## 46     48.25  57.75
## 47     56.75  61.50
## 48     59.00  49.00
## 49     86.25  28.00
## 50     92.00  23.75
## 51     98.25   8.50
## 52     93.00   3.50
## 53     98.25   3.00
## 54     93.25   1.50
## 55     80.25  16.00
## 56     83.25  15.75
## 57     82.00  10.50
## 58     92.00  29.75
## 59     89.00   3.50
## 60     89.00   4.00
## 61     78.25   5.00
## 62     93.50   6.75
## 63     93.75   1.25
## 64     92.75  10.75
## 65    104.75   9.75
## 66    104.75   3.00
## 67    103.75   2.50
## 68    115.25   4.75
## 69    104.25   2.50
## 70    111.00   1.75
## 71    117.75   1.00
## 72    106.25   0.50
```

```
## 73      50.25  67.25
## 74      54.00  52.75
## 75      61.00  49.75
## 76     122.25   4.25
## 77     105.75   1.75
## 78     116.75   1.50
## 79      59.00  58.25
## 80      64.50  52.25
## 81      75.50  39.00
## 82      99.50   2.75
## 83     104.75   2.75
## 84     115.25   2.00
## 85      52.50  56.25
## 86      95.75  16.25
## 87     102.75   3.00
## 88     118.75   1.50
## 89     108.00   1.25
## 90     106.00   0.50
## 91      67.50  43.50
## 92      71.00  35.25
## 93      84.00  26.50
## 94      77.00   1.00
## 95     107.50   1.50
## 96      98.75   0.75
## 97     110.75   2.50
## 98      96.25   1.75
## 99     105.75   1.25
## 100     93.75  17.75
## 101    107.50  10.50
## 102    116.00   8.50
## 103     82.75   2.00
## 104    120.25   2.00
## 105    105.75   1.25
## 106     85.50  13.75
## 107    110.00  15.75
## 108     95.75  13.75
```

```
Predicdrug.ll<-drm(vivosX/totalX~conc, droga, data=Predicdrug, fct = LL.5(), weights = totalX, type=("bi
op <- par(mfrow = c(1, 1))

plot(Predicdrug.ll,broken = T,xlab = "concentrations (%)",
     xlim = range(Predicdrug$conc), ylim = c(0,0.4), ylab = "Resistance (%)", col=c("red", "black","blu
     cex.legend =  0.7, lty=1, lwd=1)
```

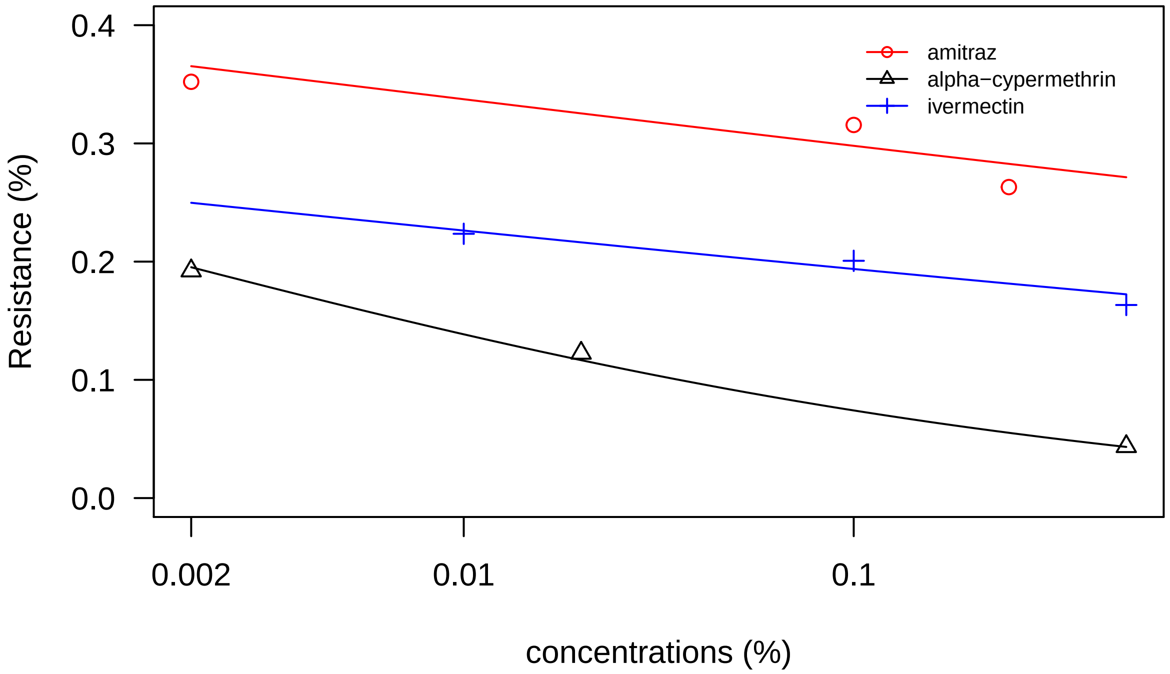
amitraz
alpha−cypermethrin
ivermectin
Resistance (%)
0.0
0.1
0.2
0.3
0.4
0.002
0.01
0.1
concentrations (%)
