# Supplementary material for: The current status of resistance to alpha-cypermethrin, ivermectin, and amitraz of the cattle tick (Rhipicephalus microplus) in Ecuador
Source: PLoS One. 2017 Apr 7;12(4):e0174652. doi: 10.1371/journal.pone.0174652 (PMC5384665; doi:10.1371/journal.pone.0174652)

Predictive_dose-response_per_farm.R

nuinuce

Sun Jan 22 19:47:36 2017

```
#Analysis were based on three different concentrations per acaricide
#i.e. alpha-cypermethrin at 0.002%, 0.02%, and 0.5%; amitraz at 0.002%, 0.1%, and 0.25% and ivermectin
#at 0.01%, 0.1%, and 0.5% for the minimum, medium or discriminatory and maximum doses, respectively.
#All farms except farm 8 showed resistance levels (more than 10%) according to use frequency of each
#acaricide

library(drc)
```

```
## Loading required package: MASS
##
## 'drc' has been loaded.
##
## Please cite R and 'drc' if used for a publication,
## for references type 'citation()' and 'citation('drc')'.
##
##
## Attaching package: 'drc'
##
## The following objects are masked from 'package:stats':
##
##     gaussian, getInitial
```

```
Predic<-read.csv("resist.csv",header = T,sep = ";", dec = ",")
Predic
```

```
##                    Localidad  finca              droga bioensayo  conc
## 1                 Los Bancos  Fin 1            amitraz       LPT 0.002
## 2                 Los Bancos  Fin 1            amitraz       LPT 0.002
## 3                 Los Bancos  Fin 1            amitraz       LIT 0.002
## 4                 Los Bancos  Fin 1            amitraz       LIT 0.002
## 5                 Los Bancos  Fin 1            amitraz       LPT 0.100
## 6                 Los Bancos  Fin 1            amitraz       LPT 0.100
## 7                 Los Bancos  Fin 1            amitraz       LIT 0.100
## 8                 Los Bancos  Fin 1            amitraz       LIT 0.100
## 9                 Los Bancos  Fin 1            amitraz       LPT 0.250
## 10                Los Bancos  Fin 1            amitraz       LPT 0.250
## 11                Los Bancos  Fin 1            amitraz       LIT 0.250
## 12                Los Bancos  Fin 1            amitraz       LIT 0.250
## 13                Los Bancos  Fin 1 alpha-cypermethrin       LPT 0.002
## 14                Los Bancos  Fin 1 alpha-cypermethrin       LPT 0.002
## 15                Los Bancos  Fin 1 alpha-cypermethrin       LIT 0.002
## 16                Los Bancos  Fin 1 alpha-cypermethrin       LIT 0.002
## 17                Los Bancos  Fin 1 alpha-cypermethrin       LPT 0.020
## 18                Los Bancos  Fin 1 alpha-cypermethrin       LPT 0.020
## 19                Los Bancos  Fin 1 alpha-cypermethrin       LIT 0.020
## 20                Los Bancos  Fin 1 alpha-cypermethrin       LIT 0.020
```

```
## 21                Los Bancos  Fin 1 alpha-cypermethrin       LPT 0.500
## 22                Los Bancos  Fin 1 alpha-cypermethrin       LPT 0.500
## 23                Los Bancos  Fin 1 alpha-cypermethrin       LIT 0.500
## 24                Los Bancos  Fin 1 alpha-cypermethrin       LIT 0.500
## 25                Los Bancos  Fin 1         ivermectin       LPT 0.010
## 26                Los Bancos  Fin 1         ivermectin       LPT 0.010
## 27                Los Bancos  Fin 1         ivermectin       LIT 0.010
## 28                Los Bancos  Fin 1         ivermectin       LIT 0.010
## 29                Los Bancos  Fin 1         ivermectin       LPT 0.100
## 30                Los Bancos  Fin 1         ivermectin       LPT 0.100
## 31                Los Bancos  Fin 1         ivermectin       LIT 0.100
## 32                Los Bancos  Fin 1         ivermectin       LIT 0.100
## 33                Los Bancos  Fin 1         ivermectin       LPT 0.500
## 34                Los Bancos  Fin 1         ivermectin       LPT 0.500
## 35                Los Bancos  Fin 1         ivermectin       LIT 0.500
## 36                Los Bancos  Fin 1         ivermectin       LIT 0.500
## 37                Los Bancos  Fin 2           amitraz        LPT 0.002
## 38                Los Bancos  Fin 2           amitraz        LPT 0.002
## 39                Los Bancos  Fin 2           amitraz        LIT 0.002
## 40                Los Bancos  Fin 2           amitraz        LIT 0.002
## 41                Los Bancos  Fin 2           amitraz        LPT 0.100
## 42                Los Bancos  Fin 2           amitraz        LPT 0.100
## 43                Los Bancos  Fin 2           amitraz        LIT 0.100
## 44                Los Bancos  Fin 2           amitraz        LIT 0.100
## 45                Los Bancos  Fin 2           amitraz        LPT 0.250
## 46                Los Bancos  Fin 2           amitraz        LPT 0.250
## 47                Los Bancos  Fin 2           amitraz        LIT 0.250
## 48                Los Bancos  Fin 2           amitraz        LIT 0.250
## 49                Los Bancos  Fin 2 alpha-cypermethrin       LPT 0.002
## 50                Los Bancos  Fin 2 alpha-cypermethrin       LPT 0.002
## 51                Los Bancos  Fin 2 alpha-cypermethrin       LIT 0.002
## 52                Los Bancos  Fin 2 alpha-cypermethrin       LIT 0.002
## 53                Los Bancos  Fin 2 alpha-cypermethrin       LPT 0.020
## 54                Los Bancos  Fin 2 alpha-cypermethrin       LPT 0.020
## 55                Los Bancos  Fin 2 alpha-cypermethrin       LIT 0.020
## 56                Los Bancos  Fin 2 alpha-cypermethrin       LIT 0.020
## 57                Los Bancos  Fin 2 alpha-cypermethrin       LPT 0.500
## 58                Los Bancos  Fin 2 alpha-cypermethrin       LPT 0.500
## 59                Los Bancos  Fin 2 alpha-cypermethrin       LIT 0.500
## 60                Los Bancos  Fin 2 alpha-cypermethrin       LIT 0.500
## 61                Los Bancos  Fin 2         ivermectin       LPT 0.010
## 62                Los Bancos  Fin 2         ivermectin       LPT 0.010
## 63                Los Bancos  Fin 2         ivermectin       LIT 0.010
## 64                Los Bancos  Fin 2         ivermectin       LIT 0.010
## 65                Los Bancos  Fin 2         ivermectin       LPT 0.100
## 66                Los Bancos  Fin 2         ivermectin       LPT 0.100
## 67                Los Bancos  Fin 2         ivermectin       LIT 0.100
## 68                Los Bancos  Fin 2         ivermectin       LIT 0.100
## 69                Los Bancos  Fin 2         ivermectin       LPT 0.500
## 70                Los Bancos  Fin 2         ivermectin       LPT 0.500
## 71                Los Bancos  Fin 2         ivermectin       LIT 0.500
## 72                Los Bancos  Fin 2         ivermectin       LIT 0.500
## 73                Los Bancos  Fin 3           amitraz        LPT 0.002
## 74                Los Bancos  Fin 3           amitraz        LPT 0.002
```

```
## 75               Los Bancos  Fin 3            amitraz       LIT 0.002
## 76               Los Bancos  Fin 3            amitraz       LIT 0.002
## 77               Los Bancos  Fin 3            amitraz       LPT 0.100
## 78               Los Bancos  Fin 3            amitraz       LPT 0.100
## 79               Los Bancos  Fin 3            amitraz       LIT 0.100
## 80               Los Bancos  Fin 3            amitraz       LIT 0.100
## 81               Los Bancos  Fin 3            amitraz       LPT 0.250
## 82               Los Bancos  Fin 3            amitraz       LPT 0.250
## 83               Los Bancos  Fin 3            amitraz       LIT 0.250
## 84               Los Bancos  Fin 3            amitraz       LIT 0.250
## 85               Los Bancos  Fin 3 alpha-cypermethrin       LPT 0.002
## 86               Los Bancos  Fin 3 alpha-cypermethrin       LPT 0.002
## 87               Los Bancos  Fin 3 alpha-cypermethrin       LIT 0.002
## 88               Los Bancos  Fin 3 alpha-cypermethrin       LIT 0.002
## 89               Los Bancos  Fin 3 alpha-cypermethrin       LPT 0.020
## 90               Los Bancos  Fin 3 alpha-cypermethrin       LPT 0.020
## 91               Los Bancos  Fin 3 alpha-cypermethrin       LIT 0.020
## 92               Los Bancos  Fin 3 alpha-cypermethrin       LIT 0.020
## 93               Los Bancos  Fin 3 alpha-cypermethrin       LPT 0.500
## 94               Los Bancos  Fin 3 alpha-cypermethrin       LPT 0.500
## 95               Los Bancos  Fin 3 alpha-cypermethrin       LIT 0.500
## 96               Los Bancos  Fin 3 alpha-cypermethrin       LIT 0.500
## 97               Los Bancos  Fin 3         ivermectin       LPT 0.010
## 98               Los Bancos  Fin 3         ivermectin       LPT 0.010
## 99               Los Bancos  Fin 3         ivermectin       LIT 0.010
## 100              Los Bancos  Fin 3         ivermectin       LIT 0.010
## 101              Los Bancos  Fin 3         ivermectin       LPT 0.100
## 102              Los Bancos  Fin 3         ivermectin       LPT 0.100
## 103              Los Bancos  Fin 3         ivermectin       LIT 0.100
## 104              Los Bancos  Fin 3         ivermectin       LIT 0.100
## 105              Los Bancos  Fin 3         ivermectin       LPT 0.500
## 106              Los Bancos  Fin 3         ivermectin       LPT 0.500
## 107              Los Bancos  Fin 3         ivermectin       LIT 0.500
## 108              Los Bancos  Fin 3         ivermectin       LIT 0.500
## 109           Santo Domingo  Fin 4            amitraz       LPT 0.002
## 110           Santo Domingo  Fin 4            amitraz       LPT 0.002
## 111           Santo Domingo  Fin 4            amitraz       LIT 0.002
## 112           Santo Domingo  Fin 4            amitraz       LIT 0.002
## 113           Santo Domingo  Fin 4            amitraz       LPT 0.100
## 114           Santo Domingo  Fin 4            amitraz       LPT 0.100
## 115           Santo Domingo  Fin 4            amitraz       LIT 0.100
## 116           Santo Domingo  Fin 4            amitraz       LIT 0.100
## 117           Santo Domingo  Fin 4            amitraz       LPT 0.250
## 118           Santo Domingo  Fin 4            amitraz       LPT 0.250
## 119           Santo Domingo  Fin 4            amitraz       LIT 0.250
## 120           Santo Domingo  Fin 4            amitraz       LIT 0.250
## 121           Santo Domingo  Fin 4 alpha-cypermethrin       LPT 0.002
## 122           Santo Domingo  Fin 4 alpha-cypermethrin       LPT 0.002
## 123           Santo Domingo  Fin 4 alpha-cypermethrin       LIT 0.002
## 124           Santo Domingo  Fin 4 alpha-cypermethrin       LIT 0.002
## 125           Santo Domingo  Fin 4 alpha-cypermethrin       LPT 0.020
## 126           Santo Domingo  Fin 4 alpha-cypermethrin       LPT 0.020
## 127           Santo Domingo  Fin 4 alpha-cypermethrin       LIT 0.020
## 128           Santo Domingo  Fin 4 alpha-cypermethrin       LIT 0.020
```

```
## 129          Santo Domingo  Fin 4 alpha-cypermethrin       LPT 0.500
## 130          Santo Domingo  Fin 4 alpha-cypermethrin       LPT 0.500
## 131          Santo Domingo  Fin 4 alpha-cypermethrin       LIT 0.500
## 132          Santo Domingo  Fin 4 alpha-cypermethrin       LIT 0.500
## 133          Santo Domingo  Fin 4         ivermectin       LPT 0.010
## 134          Santo Domingo  Fin 4         ivermectin       LPT 0.010
## 135          Santo Domingo  Fin 4         ivermectin       LIT 0.010
## 136          Santo Domingo  Fin 4         ivermectin       LIT 0.010
## 137          Santo Domingo  Fin 4         ivermectin       LPT 0.100
## 138          Santo Domingo  Fin 4         ivermectin       LPT 0.100
## 139          Santo Domingo  Fin 4         ivermectin       LIT 0.100
## 140          Santo Domingo  Fin 4         ivermectin       LIT 0.100
## 141          Santo Domingo  Fin 4         ivermectin       LPT 0.500
## 142          Santo Domingo  Fin 4         ivermectin       LPT 0.500
## 143          Santo Domingo  Fin 4         ivermectin       LIT 0.500
## 144          Santo Domingo  Fin 4         ivermectin       LIT 0.500
## 145              El Carmen  Fin 5            amitraz       LPT 0.002
## 146              El Carmen  Fin 5            amitraz       LPT 0.002
## 147              El Carmen  Fin 5            amitraz       LIT 0.002
## 148              El Carmen  Fin 5            amitraz       LIT 0.002
## 149              El Carmen  Fin 5            amitraz       LPT 0.100
## 150              El Carmen  Fin 5            amitraz       LPT 0.100
## 151              El Carmen  Fin 5            amitraz       LIT 0.100
## 152              El Carmen  Fin 5            amitraz       LIT 0.100
## 153              El Carmen  Fin 5            amitraz       LPT 0.250
## 154              El Carmen  Fin 5            amitraz       LPT 0.250
## 155              El Carmen  Fin 5            amitraz       LIT 0.250
## 156              El Carmen  Fin 5            amitraz       LIT 0.250
## 157              El Carmen  Fin 5 alpha-cypermethrin       LPT 0.002
## 158              El Carmen  Fin 5 alpha-cypermethrin       LPT 0.002
## 159              El Carmen  Fin 5 alpha-cypermethrin       LIT 0.002
## 160              El Carmen  Fin 5 alpha-cypermethrin       LIT 0.002
## 161              El Carmen  Fin 5 alpha-cypermethrin       LPT 0.020
## 162              El Carmen  Fin 5 alpha-cypermethrin       LPT 0.020
## 163              El Carmen  Fin 5 alpha-cypermethrin       LIT 0.020
## 164              El Carmen  Fin 5 alpha-cypermethrin       LIT 0.020
## 165              El Carmen  Fin 5 alpha-cypermethrin       LPT 0.500
## 166              El Carmen  Fin 5 alpha-cypermethrin       LPT 0.500
## 167              El Carmen  Fin 5 alpha-cypermethrin       LIT 0.500
## 168              El Carmen  Fin 5 alpha-cypermethrin       LIT 0.500
## 169              El Carmen  Fin 5         ivermectin       LPT 0.010
## 170              El Carmen  Fin 5         ivermectin       LPT 0.010
## 171              El Carmen  Fin 5         ivermectin       LIT 0.010
## 172              El Carmen  Fin 5         ivermectin       LIT 0.010
## 173              El Carmen  Fin 5         ivermectin       LPT 0.100
## 174              El Carmen  Fin 5         ivermectin       LPT 0.100
## 175              El Carmen  Fin 5         ivermectin       LIT 0.100
## 176              El Carmen  Fin 5         ivermectin       LIT 0.100
## 177              El Carmen  Fin 5         ivermectin       LPT 0.500
## 178              El Carmen  Fin 5         ivermectin       LPT 0.500
## 179              El Carmen  Fin 5         ivermectin       LIT 0.500
## 180              El Carmen  Fin 5         ivermectin       LIT 0.500
## 181              El Carmen  Fin 6            amitraz       LPT 0.002
## 182              El Carmen  Fin 6            amitraz       LPT 0.002
```

```
## 183                El Carmen  Fin 6            amitraz       LIT 0.002
## 184                El Carmen  Fin 6            amitraz       LIT 0.002
## 185                El Carmen  Fin 6            amitraz       LPT 0.100
## 186                El Carmen  Fin 6            amitraz       LPT 0.100
## 187                El Carmen  Fin 6            amitraz       LIT 0.100
## 188                El Carmen  Fin 6            amitraz       LIT 0.100
## 189                El Carmen  Fin 6            amitraz       LPT 0.250
## 190                El Carmen  Fin 6            amitraz       LPT 0.250
## 191                El Carmen  Fin 6            amitraz       LIT 0.250
## 192                El Carmen  Fin 6            amitraz       LIT 0.250
## 193                El Carmen  Fin 6 alpha-cypermethrin       LPT 0.002
## 194                El Carmen  Fin 6 alpha-cypermethrin       LPT 0.002
## 195                El Carmen  Fin 6 alpha-cypermethrin       LIT 0.002
## 196                El Carmen  Fin 6 alpha-cypermethrin       LIT 0.002
## 197                El Carmen  Fin 6 alpha-cypermethrin       LPT 0.020
## 198                El Carmen  Fin 6 alpha-cypermethrin       LPT 0.020
## 199                El Carmen  Fin 6 alpha-cypermethrin       LIT 0.020
## 200                El Carmen  Fin 6 alpha-cypermethrin       LIT 0.020
## 201                El Carmen  Fin 6 alpha-cypermethrin       LPT 0.500
## 202                El Carmen  Fin 6 alpha-cypermethrin       LPT 0.500
## 203                El Carmen  Fin 6 alpha-cypermethrin       LIT 0.500
## 204                El Carmen  Fin 6 alpha-cypermethrin       LIT 0.500
## 205                El Carmen  Fin 6         ivermectin       LPT 0.010
## 206                El Carmen  Fin 6         ivermectin       LPT 0.010
## 207                El Carmen  Fin 6         ivermectin       LIT 0.010
## 208                El Carmen  Fin 6         ivermectin       LIT 0.010
## 209                El Carmen  Fin 6         ivermectin       LPT 0.100
## 210                El Carmen  Fin 6         ivermectin       LPT 0.100
## 211                El Carmen  Fin 6         ivermectin       LIT 0.100
## 212                El Carmen  Fin 6         ivermectin       LIT 0.100
## 213                El Carmen  Fin 6         ivermectin       LPT 0.500
## 214                El Carmen  Fin 6         ivermectin       LPT 0.500
## 215                El Carmen  Fin 6         ivermectin       LIT 0.500
## 216                El Carmen  Fin 6         ivermectin       LIT 0.500
## 217                El Carmen  Fin 7            amitraz       LPT 0.002
## 218                El Carmen  Fin 7            amitraz       LPT 0.002
## 219                El Carmen  Fin 7            amitraz       LIT 0.002
## 220                El Carmen  Fin 7            amitraz       LIT 0.002
## 221                El Carmen  Fin 7            amitraz       LPT 0.100
## 222                El Carmen  Fin 7            amitraz       LPT 0.100
## 223                El Carmen  Fin 7            amitraz       LIT 0.100
## 224                El Carmen  Fin 7            amitraz       LIT 0.100
## 225                El Carmen  Fin 7            amitraz       LPT 0.250
## 226                El Carmen  Fin 7            amitraz       LPT 0.250
## 227                El Carmen  Fin 7            amitraz       LIT 0.250
## 228                El Carmen  Fin 7            amitraz       LIT 0.250
## 229                El Carmen  Fin 7 alpha-cypermethrin       LPT 0.002
## 230                El Carmen  Fin 7 alpha-cypermethrin       LPT 0.002
## 231                El Carmen  Fin 7 alpha-cypermethrin       LIT 0.002
## 232                El Carmen  Fin 7 alpha-cypermethrin       LIT 0.002
## 233                El Carmen  Fin 7 alpha-cypermethrin       LPT 0.020
## 234                El Carmen  Fin 7 alpha-cypermethrin       LPT 0.020
## 235                El Carmen  Fin 7 alpha-cypermethrin       LIT 0.020
## 236                El Carmen  Fin 7 alpha-cypermethrin       LIT 0.020
```

```
## 237             El Carmen  Fin 7 alpha-cypermethrin       LPT 0.500
## 238             El Carmen  Fin 7 alpha-cypermethrin       LPT 0.500
## 239             El Carmen  Fin 7 alpha-cypermethrin       LIT 0.500
## 240             El Carmen  Fin 7 alpha-cypermethrin       LIT 0.500
## 241             El Carmen  Fin 7         ivermectin       LPT 0.010
## 242             El Carmen  Fin 7         ivermectin       LPT 0.010
## 243             El Carmen  Fin 7         ivermectin       LIT 0.010
## 244             El Carmen  Fin 7         ivermectin       LIT 0.010
## 245             El Carmen  Fin 7         ivermectin       LPT 0.100
## 246             El Carmen  Fin 7         ivermectin       LPT 0.100
## 247             El Carmen  Fin 7         ivermectin       LIT 0.100
## 248             El Carmen  Fin 7         ivermectin       LIT 0.100
## 249             El Carmen  Fin 7         ivermectin       LPT 0.500
## 250             El Carmen  Fin 7         ivermectin       LPT 0.500
## 251             El Carmen  Fin 7         ivermectin       LIT 0.500
## 252             El Carmen  Fin 7         ivermectin       LIT 0.500
## 253         Santo Domingo  Fin 8           amitraz       LPT 0.002
## 254         Santo Domingo  Fin 8           amitraz       LPT 0.002
## 255         Santo Domingo  Fin 8           amitraz       LIT 0.002
## 256         Santo Domingo  Fin 8           amitraz       LIT 0.002
## 257         Santo Domingo  Fin 8           amitraz       LPT 0.100
## 258         Santo Domingo  Fin 8           amitraz       LPT 0.100
## 259         Santo Domingo  Fin 8           amitraz       LIT 0.100
## 260         Santo Domingo  Fin 8           amitraz       LIT 0.100
## 261         Santo Domingo  Fin 8           amitraz       LPT 0.250
## 262         Santo Domingo  Fin 8           amitraz       LPT 0.250
## 263         Santo Domingo  Fin 8           amitraz       LIT 0.250
## 264         Santo Domingo  Fin 8           amitraz       LIT 0.250
## 265         Santo Domingo  Fin 8 alpha-cypermethrin       LPT 0.002
## 266         Santo Domingo  Fin 8 alpha-cypermethrin       LPT 0.002
## 267         Santo Domingo  Fin 8 alpha-cypermethrin       LIT 0.002
## 268         Santo Domingo  Fin 8 alpha-cypermethrin       LIT 0.002
## 269         Santo Domingo  Fin 8 alpha-cypermethrin       LPT 0.020
## 270         Santo Domingo  Fin 8 alpha-cypermethrin       LPT 0.020
## 271         Santo Domingo  Fin 8 alpha-cypermethrin       LIT 0.020
## 272         Santo Domingo  Fin 8 alpha-cypermethrin       LIT 0.020
## 273         Santo Domingo  Fin 8 alpha-cypermethrin       LPT 0.500
## 274         Santo Domingo  Fin 8 alpha-cypermethrin       LPT 0.500
## 275         Santo Domingo  Fin 8 alpha-cypermethrin       LIT 0.500
## 276         Santo Domingo  Fin 8 alpha-cypermethrin       LIT 0.500
## 277         Santo Domingo  Fin 8         ivermectin       LPT 0.010
## 278         Santo Domingo  Fin 8         ivermectin       LPT 0.010
## 279         Santo Domingo  Fin 8         ivermectin       LIT 0.010
## 280         Santo Domingo  Fin 8         ivermectin       LIT 0.010
## 281         Santo Domingo  Fin 8         ivermectin       LPT 0.100
## 282         Santo Domingo  Fin 8         ivermectin       LPT 0.100
## 283         Santo Domingo  Fin 8         ivermectin       LIT 0.100
## 284         Santo Domingo  Fin 8         ivermectin       LIT 0.100
## 285         Santo Domingo  Fin 8         ivermectin       LPT 0.500
## 286         Santo Domingo  Fin 8         ivermectin       LPT 0.500
## 287         Santo Domingo  Fin 8         ivermectin       LIT 0.500
## 288         Santo Domingo  Fin 8         ivermectin       LIT 0.500
## 289         Santo Domingo  Fin 9           amitraz       LPT 0.002
## 290         Santo Domingo  Fin 9           amitraz       LPT 0.002
```

```
## 291           Santo Domingo  Fin 9            amitraz       LIT 0.002
## 292           Santo Domingo  Fin 9            amitraz       LIT 0.002
## 293           Santo Domingo  Fin 9            amitraz       LPT 0.100
## 294           Santo Domingo  Fin 9            amitraz       LPT 0.100
## 295           Santo Domingo  Fin 9            amitraz       LIT 0.100
## 296           Santo Domingo  Fin 9            amitraz       LIT 0.100
## 297           Santo Domingo  Fin 9            amitraz       LPT 0.250
## 298           Santo Domingo  Fin 9            amitraz       LPT 0.250
## 299           Santo Domingo  Fin 9            amitraz       LIT 0.250
## 300           Santo Domingo  Fin 9            amitraz       LIT 0.250
## 301           Santo Domingo  Fin 9 alpha-cypermethrin       LPT 0.002
## 302           Santo Domingo  Fin 9 alpha-cypermethrin       LPT 0.002
## 303           Santo Domingo  Fin 9 alpha-cypermethrin       LIT 0.002
## 304           Santo Domingo  Fin 9 alpha-cypermethrin       LIT 0.002
## 305           Santo Domingo  Fin 9 alpha-cypermethrin       LPT 0.020
## 306           Santo Domingo  Fin 9 alpha-cypermethrin       LPT 0.020
## 307           Santo Domingo  Fin 9 alpha-cypermethrin       LIT 0.020
## 308           Santo Domingo  Fin 9 alpha-cypermethrin       LIT 0.020
## 309           Santo Domingo  Fin 9 alpha-cypermethrin       LPT 0.500
## 310           Santo Domingo  Fin 9 alpha-cypermethrin       LPT 0.500
## 311           Santo Domingo  Fin 9 alpha-cypermethrin       LIT 0.500
## 312           Santo Domingo  Fin 9 alpha-cypermethrin       LIT 0.500
## 313           Santo Domingo  Fin 9         ivermectin       LPT 0.010
## 314           Santo Domingo  Fin 9         ivermectin       LPT 0.010
## 315           Santo Domingo  Fin 9         ivermectin       LIT 0.010
## 316           Santo Domingo  Fin 9         ivermectin       LIT 0.010
## 317           Santo Domingo  Fin 9         ivermectin       LPT 0.100
## 318           Santo Domingo  Fin 9         ivermectin       LPT 0.100
## 319           Santo Domingo  Fin 9         ivermectin       LIT 0.100
## 320           Santo Domingo  Fin 9         ivermectin       LIT 0.100
## 321           Santo Domingo  Fin 9         ivermectin       LPT 0.500
## 322           Santo Domingo  Fin 9         ivermectin       LPT 0.500
## 323           Santo Domingo  Fin 9         ivermectin       LIT 0.500
## 324           Santo Domingo  Fin 9         ivermectin       LIT 0.500
## 325 Pedro Vicente Maldonado Fin 10            amitraz       LPT 0.002
## 326 Pedro Vicente Maldonado Fin 10            amitraz       LPT 0.002
## 327 Pedro Vicente Maldonado Fin 10            amitraz       LIT 0.002
## 328 Pedro Vicente Maldonado Fin 10            amitraz       LIT 0.002
## 329 Pedro Vicente Maldonado Fin 10            amitraz       LPT 0.100
## 330 Pedro Vicente Maldonado Fin 10            amitraz       LPT 0.100
## 331 Pedro Vicente Maldonado Fin 10            amitraz       LIT 0.100
## 332 Pedro Vicente Maldonado Fin 10            amitraz       LIT 0.100
## 333 Pedro Vicente Maldonado Fin 10            amitraz       LPT 0.250
## 334 Pedro Vicente Maldonado Fin 10            amitraz       LPT 0.250
## 335 Pedro Vicente Maldonado Fin 10            amitraz       LIT 0.250
## 336 Pedro Vicente Maldonado Fin 10            amitraz       LIT 0.250
## 337 Pedro Vicente Maldonado Fin 10 alpha-cypermethrin       LPT 0.002
## 338 Pedro Vicente Maldonado Fin 10 alpha-cypermethrin       LPT 0.002
## 339 Pedro Vicente Maldonado Fin 10 alpha-cypermethrin       LIT 0.002
## 340 Pedro Vicente Maldonado Fin 10 alpha-cypermethrin       LIT 0.002
## 341 Pedro Vicente Maldonado Fin 10 alpha-cypermethrin       LPT 0.020
## 342 Pedro Vicente Maldonado Fin 10 alpha-cypermethrin       LPT 0.020
## 343 Pedro Vicente Maldonado Fin 10 alpha-cypermethrin       LIT 0.020
## 344 Pedro Vicente Maldonado Fin 10 alpha-cypermethrin       LIT 0.020
```

```
## 345 Pedro Vicente Maldonado Fin 10 alpha-cypermethrin       LPT 0.500
## 346 Pedro Vicente Maldonado Fin 10 alpha-cypermethrin       LPT 0.500
## 347 Pedro Vicente Maldonado Fin 10 alpha-cypermethrin       LIT 0.500
## 348 Pedro Vicente Maldonado Fin 10 alpha-cypermethrin       LIT 0.500
## 349 Pedro Vicente Maldonado Fin 10         ivermectin       LPT 0.010
## 350 Pedro Vicente Maldonado Fin 10         ivermectin       LPT 0.010
## 351 Pedro Vicente Maldonado Fin 10         ivermectin       LIT 0.010
## 352 Pedro Vicente Maldonado Fin 10         ivermectin       LIT 0.010
## 353 Pedro Vicente Maldonado Fin 10         ivermectin       LPT 0.100
## 354 Pedro Vicente Maldonado Fin 10         ivermectin       LPT 0.100
## 355 Pedro Vicente Maldonado Fin 10         ivermectin       LIT 0.100
## 356 Pedro Vicente Maldonado Fin 10         ivermectin       LIT 0.100
## 357 Pedro Vicente Maldonado Fin 10         ivermectin       LPT 0.500
## 358 Pedro Vicente Maldonado Fin 10         ivermectin       LPT 0.500
## 359 Pedro Vicente Maldonado Fin 10         ivermectin       LIT 0.500
## 360 Pedro Vicente Maldonado Fin 10         ivermectin       LIT 0.500
## 361 Pedro Vicente Maldonado Fin 11            amitraz       LPT 0.002
## 362 Pedro Vicente Maldonado Fin 11            amitraz       LPT 0.002
## 363 Pedro Vicente Maldonado Fin 11            amitraz       LIT 0.002
## 364 Pedro Vicente Maldonado Fin 11            amitraz       LIT 0.002
## 365 Pedro Vicente Maldonado Fin 11            amitraz       LPT 0.100
## 366 Pedro Vicente Maldonado Fin 11            amitraz       LPT 0.100
## 367 Pedro Vicente Maldonado Fin 11            amitraz       LIT 0.100
## 368 Pedro Vicente Maldonado Fin 11            amitraz       LIT 0.100
## 369 Pedro Vicente Maldonado Fin 11            amitraz       LPT 0.250
## 370 Pedro Vicente Maldonado Fin 11            amitraz       LPT 0.250
## 371 Pedro Vicente Maldonado Fin 11            amitraz       LIT 0.250
## 372 Pedro Vicente Maldonado Fin 11            amitraz       LIT 0.250
## 373 Pedro Vicente Maldonado Fin 11 alpha-cypermethrin       LPT 0.002
## 374 Pedro Vicente Maldonado Fin 11 alpha-cypermethrin       LPT 0.002
## 375 Pedro Vicente Maldonado Fin 11 alpha-cypermethrin       LIT 0.002
## 376 Pedro Vicente Maldonado Fin 11 alpha-cypermethrin       LIT 0.002
## 377 Pedro Vicente Maldonado Fin 11 alpha-cypermethrin       LPT 0.020
## 378 Pedro Vicente Maldonado Fin 11 alpha-cypermethrin       LPT 0.020
## 379 Pedro Vicente Maldonado Fin 11 alpha-cypermethrin       LIT 0.020
## 380 Pedro Vicente Maldonado Fin 11 alpha-cypermethrin       LIT 0.020
## 381 Pedro Vicente Maldonado Fin 11 alpha-cypermethrin       LPT 0.500
## 382 Pedro Vicente Maldonado Fin 11 alpha-cypermethrin       LPT 0.500
## 383 Pedro Vicente Maldonado Fin 11 alpha-cypermethrin       LIT 0.500
## 384 Pedro Vicente Maldonado Fin 11 alpha-cypermethrin       LIT 0.500
## 385 Pedro Vicente Maldonado Fin 11         ivermectin       LPT 0.010
## 386 Pedro Vicente Maldonado Fin 11         ivermectin       LPT 0.010
## 387 Pedro Vicente Maldonado Fin 11         ivermectin       LIT 0.010
## 388 Pedro Vicente Maldonado Fin 11         ivermectin       LIT 0.010
## 389 Pedro Vicente Maldonado Fin 11         ivermectin       LPT 0.100
## 390 Pedro Vicente Maldonado Fin 11         ivermectin       LPT 0.100
## 391 Pedro Vicente Maldonado Fin 11         ivermectin       LIT 0.100
## 392 Pedro Vicente Maldonado Fin 11         ivermectin       LIT 0.100
## 393 Pedro Vicente Maldonado Fin 11         ivermectin       LPT 0.500
## 394 Pedro Vicente Maldonado Fin 11         ivermectin       LPT 0.500
## 395 Pedro Vicente Maldonado Fin 11         ivermectin       LIT 0.500
## 396 Pedro Vicente Maldonado Fin 11         ivermectin       LIT 0.500
## 397 Pedro Vicente Maldonado Fin 12            amitraz       LPT 0.002
## 398 Pedro Vicente Maldonado Fin 12            amitraz       LPT 0.002
```

```
## 399 Pedro Vicente Maldonado Fin 12            amitraz       LIT 0.002
## 400 Pedro Vicente Maldonado Fin 12            amitraz       LIT 0.002
## 401 Pedro Vicente Maldonado Fin 12            amitraz       LPT 0.100
## 402 Pedro Vicente Maldonado Fin 12            amitraz       LPT 0.100
## 403 Pedro Vicente Maldonado Fin 12            amitraz       LIT 0.100
## 404 Pedro Vicente Maldonado Fin 12            amitraz       LIT 0.100
## 405 Pedro Vicente Maldonado Fin 12            amitraz       LPT 0.250
## 406 Pedro Vicente Maldonado Fin 12            amitraz       LPT 0.250
## 407 Pedro Vicente Maldonado Fin 12            amitraz       LIT 0.250
## 408 Pedro Vicente Maldonado Fin 12            amitraz       LIT 0.250
## 409 Pedro Vicente Maldonado Fin 12 alpha-cypermethrin       LPT 0.002
## 410 Pedro Vicente Maldonado Fin 12 alpha-cypermethrin       LPT 0.002
## 411 Pedro Vicente Maldonado Fin 12 alpha-cypermethrin       LIT 0.002
## 412 Pedro Vicente Maldonado Fin 12 alpha-cypermethrin       LIT 0.002
## 413 Pedro Vicente Maldonado Fin 12 alpha-cypermethrin       LPT 0.020
## 414 Pedro Vicente Maldonado Fin 12 alpha-cypermethrin       LPT 0.020
## 415 Pedro Vicente Maldonado Fin 12 alpha-cypermethrin       LIT 0.020
## 416 Pedro Vicente Maldonado Fin 12 alpha-cypermethrin       LIT 0.020
## 417 Pedro Vicente Maldonado Fin 12 alpha-cypermethrin       LPT 0.500
## 418 Pedro Vicente Maldonado Fin 12 alpha-cypermethrin       LPT 0.500
## 419 Pedro Vicente Maldonado Fin 12 alpha-cypermethrin       LIT 0.500
## 420 Pedro Vicente Maldonado Fin 12 alpha-cypermethrin       LIT 0.500
## 421 Pedro Vicente Maldonado Fin 12         ivermectin       LPT 0.010
## 422 Pedro Vicente Maldonado Fin 12         ivermectin       LPT 0.010
## 423 Pedro Vicente Maldonado Fin 12         ivermectin       LIT 0.010
## 424 Pedro Vicente Maldonado Fin 12         ivermectin       LIT 0.010
## 425 Pedro Vicente Maldonado Fin 12         ivermectin       LPT 0.100
## 426 Pedro Vicente Maldonado Fin 12         ivermectin       LPT 0.100
## 427 Pedro Vicente Maldonado Fin 12         ivermectin       LIT 0.100
## 428 Pedro Vicente Maldonado Fin 12         ivermectin       LIT 0.100
## 429 Pedro Vicente Maldonado Fin 12         ivermectin       LPT 0.500
## 430 Pedro Vicente Maldonado Fin 12         ivermectin       LPT 0.500
## 431 Pedro Vicente Maldonado Fin 12         ivermectin       LIT 0.500
## 432 Pedro Vicente Maldonado Fin 12         ivermectin       LIT 0.500
##     resist mort total muertos vivos
## 1       57   43   101      45    56
## 2       51   49   100      50    50
## 3       60   40    99      40    59
## 4       58   42   102      44    58
## 5       45   55    98      55    43
## 6       44   56   100      57    43
## 7       45   55    99      54    45
## 8       50   50    98      50    48
## 9       30   70    99      70    29
## 10      28   72   100      73    27
## 11      27   73   103      75    28
## 12      28   72    99      72    27
## 13      13   87   105      92    13
## 14       9   91   100      91     9
## 15      10   90   101      91    10
## 16       5   95    99      94     5
## 17      14   86   103      89    14
## 18      10   90   100      90    10
## 19       5   95    98      93     5
```

```
## 20          12   88   101       89    12
## 21           1  100   120      120     0
## 22           1  100   110      110     0
## 23           4   96   103       99     4
## 24           2   98    97       95     2
## 25          44   56    99       56    43
## 26          44   56   106       60    46
## 27          49   51   101       52    49
## 28          31   69    98       68    30
## 29          39   61   100       62    38
## 30          35   65    55       36    19
## 31          37   63   102       64    38
## 32          31   69    96       67    29
## 33          31   69   101       70    31
## 34          32   68    57       39    18
## 35          32   68    99       67    32
## 36          30   70   101       71    30
## 37           4   96   101       97     4
## 38           1  100    60       60     0
## 39           3   97    98       95     3
## 40           3   97    99       96     3
## 41           6   94   103       97     6
## 42           6   94   101       95     6
## 43           4   96   101       97     4
## 44           6   94   101       95     6
## 45           6   94   101       95     6
## 46           1  100   102      102     0
## 47           2   98    99       97     2
## 48           3   97   102       99     3
## 49          34   66    98       65    33
## 50          33   67   150      102    48
## 51          38   62    98       62    36
## 52          39   61    96       59    37
## 53          28   72   117       85    32
## 54          17   83   105       87    18
## 55          19   81   102       83    19
## 56          15   85    96       82    14
## 57          28   72   124       90    34
## 58          17   83   116       97    19
## 59           4   96    96       92     4
## 60           7   93    89       83     6
## 61          46   54    99       54    45
## 62          50   50   121       62    59
## 63          50   50    95       49    46
## 64          41   59    85       51    34
## 65          36   64   101       65    36
## 66          47   53   120       64    56
## 67          50   50    94       48    46
## 68          39   61    92       57    35
## 69          35   65    95       62    33
## 70          33   67    40       27    13
## 71          28   72    89       65    24
## 72          37   63    98       62    36
## 73          81   19   100       20    80
```

```
## 74        88   12   122        16   106
## 75        85   15   106        16    90
## 76        94    6   115         8   107
## 77        74   26   120        32    88
## 78        90   10   142        16   126
## 79        85   15    96        14    82
## 80        88   12   120        16   104
## 81        73   27   115        32    83
## 82        81   19   126        25   101
## 83        76   24   104        25    79
## 84        84   16   135        23   112
## 85         6   94   105        99     6
## 86         7   93   102        95     7
## 87         7   93   110       102     8
## 88         3   97   120       116     4
## 89         2   98   105       103     2
## 90         2   98   120       118     2
## 91         4   96   120       115     5
## 92         7   93   150       139    11
## 93         3   97   120       116     4
## 94         2   98   130       127     3
## 95         6   94    49        46     3
## 96         6   94   110       104     6
## 97        94    6   105         7    98
## 98        78   22    99        23    76
## 99        95    5    98         5    93
## 100       93    7    95         8    87
## 101       81   19   100        20    80
## 102       76   24   100        25    75
## 103       86   14   115        16    99
## 104       80   20   120        25    95
## 105       75   25   135        35   100
## 106       68   32   102        33    69
## 107       75   25   100        25    75
## 108       73   27   120        34    86
## 109       50   50   101        51    50
## 110       51   49    98        49    49
## 111       44   56    85        48    37
## 112       49   51    95        49    46
## 113       50   50    99        50    49
## 114       48   52    97        51    46
## 115       52   48    89        43    46
## 116       51   49    96        48    48
## 117       49   51   109        56    53
## 118       48   52    99        52    47
## 119       58   42    96        41    55
## 120       51   49    85        42    43
## 121       56   44   102        45    57
## 122       51   49   105        52    53
## 123       57   43    84        37    47
## 124       51   49    79        39    40
## 125       54   46    98        46    52
## 126       53   47    85        41    44
## 127       59   41    96        40    56
```

```
## 128     52   48    96      47    49
## 129      5   95   100      95     5
## 130      1   99    80      79     1
## 131      3   97    89      86     3
## 132      4   96    92      88     4
## 133      2   98   102     100     2
## 134      4   96   109     105     4
## 135      2   98    96      94     2
## 136      1   99    83      82     1
## 137      2   98   103     101     2
## 138      3   97   105     102     3
## 139      5   95   101      96     5
## 140      2   98    86      84     2
## 141      1  100   110     110     0
## 142      3   97    78      76     2
## 143      2   98    96      94     2
## 144      3   97    89      86     3
## 145     45   55    98      55    43
## 146     25   75    95      72    23
## 147     30   70    98      69    29
## 148     35   65    92      60    32
## 149     26   74   101      75    26
## 150     26   74    96      71    25
## 151     25   75    96      72    24
## 152     20   80    87      70    17
## 153      9   91    95      87     8
## 154     23   77    84      65    19
## 155     23   77   101      78    23
## 156     24   76    98      75    23
## 157     21   79    88      70    18
## 158     20   80   104      84    20
## 159     16   84    85      72    13
## 160     16   84   101      85    16
## 161      7   93   116     108     8
## 162     20   80   103      82    21
## 163     16   84    89      75    14
## 164      6   94    96      90     6
## 165      6   94   101      95     6
## 166      9   91    90      82     8
## 167     13   87    87      76    11
## 168      5   95    97      92     5
## 169     14   86   104      90    14
## 170     13   87   108      94    14
## 171      8   92    92      85     7
## 172      9   91    86      78     8
## 173     12   88    98      86    12
## 174      9   91   112     102    10
## 175      8   92    86      79     7
## 176      7   93    85      79     6
## 177      5   95   102      97     5
## 178      8   92    98      90     8
## 179      4   96    95      91     4
## 180      8   92    98      90     8
## 181     56   44   101      45    56
```

```
## 182      60   40   120        50    70
## 183      53   47    98        47    51
## 184      52   48   105        51    54
## 185      53   47   110        52    58
## 186      59   41   142        60    82
## 187      49   51   101        52    49
## 188      48   52   120        63    57
## 189      51   49   115        57    58
## 190      42   58   106        62    44
## 191      51   49   116        58    58
## 192      38   62    95        59    36
## 193      29   71   122        87    35
## 194      29   71   105        75    30
## 195      22   78   105        82    23
## 196      19   81   125       101    24
## 197      27   73   102        75    27
## 198      21   79   126       100    26
## 199      21   79   120        95    25
## 200      15   85   115        98    17
## 201       8   92    98        90     8
## 202      11   89   112       100    12
## 203       4   96    99        95     4
## 204       9   91   118       108    10
## 205       6   94   104        98     6
## 206       3   97    98        95     3
## 207       4   96    86        83     3
## 208       2   98    98        96     2
## 209       3   97   120       117     3
## 210       6   94   114       107     7
## 211       2   98    96        94     2
## 212       1  100    75        75     0
## 213       3   97   102        99     3
## 214       1   99    99        98     1
## 215       1   99    89        88     1
## 216       1   99    89        88     1
## 217      39   61    95        78    17
## 218      16   84   106        89    17
## 219      16   84    89        75    14
## 220      17   83    95        79    16
## 221      22   78    86        73    13
## 222      11   89   129       108    21
## 223      16   84    98        82    16
## 224      16   84    83        70    13
## 225      11   89    80        71     9
## 226      18   82   110        96    14
## 227      10   90    94        85     9
## 228      17   83    86        76    10
## 229      21   79    99        78    21
## 230      27   73   137       100    37
## 231      24   76   102        78    24
## 232      26   74   149       112    37
## 233       6   94   101        95     6
## 234       2   98    93        91     2
## 235       4   96    89        85     4
```

```
## 236        2   98    87        85      2
## 237        1  100    99        99      0
## 238        1   99    90        89      1
## 239        6   94    78        73      5
## 240       10   90   105        95     10
## 241        7   93    90        84      6
## 242        1   99    90        89      1
## 243       12   88    68        60      8
## 244        6   94    85        80      5
## 245        1   99   100        96      4
## 246        9   91   108        98     10
## 247        7   93    98        91      7
## 248        7   93    95        89      6
## 249        1  100   100       100      0
## 250        1  100    98        98      0
## 251        3   97    86        83      3
## 252        2   98    96        94      2
## 253       19   81   105        95     15
## 254        4   96    96        90      6
## 255       16   84   102        90     12
## 256        9   91   106        96     10
## 257        7   93    96        89      7
## 258        7   93   110       102      8
## 259       13   87   124       108     16
## 260        6   94   128       120      8
## 261        2   98   102       100      2
## 262        2   98   125       122      3
## 263        6   94   106       100      6
## 264        1   99    98        97      1
## 265        4   96   125       120      5
## 266        2   98    96        94      2
## 267        1   99    96        95      1
## 268        2   98   108       106      2
## 269        2   98   115       113      2
## 270        6   94   116       109      7
## 271        5   95   125       119      6
## 272        3   97   124       120      4
## 273        1   99   105       104      1
## 274        1  100   103       103      0
## 275        3   97   103       100      3
## 276        5   95   116       110      6
## 277        1   99   120       119      1
## 278        3   97    98        95      3
## 279        2   98   114       112      2
## 280        1   99   119       118      1
## 281        1   99   115       114      1
## 282        1  100   125       125      0
## 283        1   99   130       129      1
## 284        2   98   105       103      2
## 285        1   99   105       104      1
## 286        1  100    96        96      0
## 287        1  100   102       102      0
## 288        1   99   124       123      1
## 289       51   49   105        52     53
```

```
## 290     63  37  120   45  75
## 291     56  44  120   55  65
## 292     61  39  125   49  76
## 293     45  55  112   62  50
## 294     54  46  106   49  57
## 295     48  52  105   56  49
## 296     53  47  104   49  55
## 297     39  61  106   65  41
## 298     44  56   99   56  43
## 299     48  52  132   71  61
## 300     51  49  106   52  54
## 301      7  93  150  140  10
## 302      3  97   96   93   3
## 303      2  98  125  123   2
## 304      1  99  135  133   2
## 305      5  95  100   95   5
## 306      1  99  120  119   1
## 307      1  99  105  104   1
## 308      1 100  105  105   0
## 309      4  96  100   96   4
## 310      1 100  132  132   0
## 311      1 100   99   99   0
## 312      1  99  142  140   2
## 313     51  49  120   60  60
## 314     53  47  115   55  60
## 315     49  51  132   69  63
## 316     50  50  102   52  50
## 317     47  53  130   70  60
## 318     44  56   96   54  42
## 319     40  60  106   65  41
## 320     49  51  135   69  66
## 321     32  68  110   75  35
## 322     29  71  105   75  30
## 323     39  61  119   74  45
## 324     37  63  124   78  46
## 325      3  97  102   99   3
## 326      4  96  103   99   4
## 327      2  98  102  100   2
## 328      2  98  102  100   2
## 329      4  96  102   98   4
## 330      1 100  102  102   0
## 331      2  98  106  104   2
## 332      4  96  120  115   5
## 333      1  99  101  100   1
## 334      3  97  105  102   3
## 335      3  97  123  119   4
## 336      1 100  140  140   0
## 337     64  36  105   39  66
## 338     44  56  105   60  45
## 339     55  45  110   49  61
## 340     47  53  115   62  53
## 341      7  93   96   89   7
## 342     10  90  100   90  10
## 343     15  85  120  102  18
```

```
## 344     23   77   132     102    30
## 345      1  100   120     120     0
## 346      2   98   108     106     2
## 347      6   94    96      90     6
## 348      4   96    99      95     4
## 349      1   99    98      97     1
## 350      1  100    89      89     0
## 351      1   99   152     150     2
## 352      2   98   142     139     3
## 353      1  100   101     101     0
## 354      2   98   102     100     2
## 355      1   99   132     131     1
## 356      2   98   102     100     2
## 357      1  100    96      96     0
## 358      1  100    89      89     0
## 359      1  100   116     116     0
## 360      2   98   125     123     2
## 361     38   62   105      66    39
## 362     43   57    85      49    36
## 363     35   65   115      75    40
## 364     42   58   139      80    59
## 365     29   71   100      72    28
## 366     35   65    69      45    24
## 367     38   62   124      78    46
## 368     33   67   132      89    43
## 369     18   82   100      82    18
## 370     28   72    95      69    26
## 371     27   73   135      99    36
## 372     23   77   112      86    26
## 373      3   97    30      29     1
## 374      1  100    65      65     0
## 375      2   98   121     119     2
## 376      1   99    96      95     1
## 377      2   98   101      99     2
## 378      2   98    85      83     2
## 379      2   98   135     133     2
## 380      1  100   115     115     0
## 381      2   98   102     100     2
## 382      1  100    78      78     0
## 383      1   99    98      97     1
## 384      1  100   120     120     0
## 385      4   96   103      99     4
## 386      1   99    96      95     1
## 387      3   97   129     125     4
## 388      1   99   125     124     1
## 389      2   98   101      99     2
## 390      1  100    85      85     0
## 391      2   98    98      96     2
## 392      3   97   108     105     3
## 393      1  100   120     120     0
## 394      1   99    88      87     1
## 395      1   99   100      99     1
## 396      2   98   120     117     3
## 397     17   83    96      80    16
```

```
## 398      8   92   120     100     20
## 399     16   84   125     105     20
## 400     14   86   105      90     15
## 401      7   93   124     115      9
## 402      9   91   112     102     10
## 403     19   81   124     111     13
## 404     18   82   112     102     10
## 405     11   89   116     107      9
## 406      4   96   105      99      6
## 407      7   93   142     132     10
## 408      7   93   135     126      9
## 409      5   95    20      19      1
## 410      2   98   125     123      2
## 411      3   97    95      92      3
## 412      2   98    99      97      2
## 413      3   97   110     107      3
## 414      2   98   132     130      2
## 415      1   99   105     104      1
## 416      1   99   142     140      2
## 417      3   97   120     117      3
## 418      1   99   101     100      1
## 419      1  100    98      98      0
## 420      1   99   109     108      1
## 421     20   80    98      83     15
## 422     18   82    96      83     13
## 423     12   88    89      76     13
## 424     11   89   114     100     14
## 425     13   87   115     100     15
## 426     17   83   158     140     18
## 427      6   94   102      90     12
## 428     14   86   128     110     18
## 429     11   89   102      91     11
## 430     18   82    95      82     13
## 431     15   85   135     115     20
## 432      7   93   106      95     11
```

```
head(Predic)
```

```
##    Localidad finca   droga bioensayo  conc resist mort total muertos vivos
## 1 Los Bancos Fin 1 amitraz       LPT 0.002     57   43   101      45    56
## 2 Los Bancos Fin 1 amitraz       LPT 0.002     51   49   100      50    50
## 3 Los Bancos Fin 1 amitraz       LIT 0.002     60   40    99      40    59
## 4 Los Bancos Fin 1 amitraz       LIT 0.002     58   42   102      44    58
## 5 Los Bancos Fin 1 amitraz       LPT 0.100     45   55    98      55    43
## 6 Los Bancos Fin 1 amitraz       LPT 0.100     44   56   100      57    43
```

```
fin1<-subset(Predic, finca=="Fin 1")
f1a.ll<-drm(vivos/total~conc, droga, data=fin1,fct = LL.4(), weights = total, type=("binomial"))
fin2<-subset(Predic, finca=="Fin 2")
f2a.ll<-drm(vivos/total~conc, droga, data=fin2,fct = LL.4(), weights = total, type=("binomial"))
fin3<-subset(Predic, finca=="Fin 3")
f3a.ll<-drm(vivos/total~conc, droga, data=fin3,fct = LL.4(), weights = total, type=("binomial"))
fin4<-subset(Predic, finca=="Fin 4")
```

```
f4a.ll<-drm(vivos/total~conc, droga,data=fin4,fct = LL.4(), weights = total, type=("binomial"))
fin5<-subset(Predic, finca=="Fin 5")
f5a.ll<-drm(vivos/total~conc, droga, data=fin5,fct = LL.4(), weights = total, type=("binomial"))
fin6<-subset(Predic, finca=="Fin 6")
f6a.ll<-drm(vivos/total~conc, droga, data=fin6,fct = LL.4(), weights = total, type=("binomial"))
fin7<-subset(Predic, finca=="Fin 7")
f7a.ll<-drm(vivos/total~conc, droga,data=fin7,fct = LL.3(), weights = total, type=("binomial"))
fin8<-subset(Predic, finca=="Fin 8")
f8a.ll<-drm(vivos/total~conc, droga,data=fin8,fct = LL.4(), weights = total, type=("binomial"))
fin9<-subset(Predic, finca=="Fin 9")
f9a.ll<-drm(vivos/total~conc,droga, data=fin9,fct = LL.4(), weights = total, type=("binomial"))
fin10<-subset(Predic, finca=="Fin 10")
f10a.ll<-drm(vivos/total~conc, droga, data=fin10,fct = LL.4(), weights = total, type=("binomial"))
fin11<-subset(Predic, finca=="Fin 11")
f11a.ll<-drm(vivos/total~conc, droga, data=fin11,fct = LL.4(), weights = total, type=("binomial"))
fin12<-subset(Predic, finca=="Fin 12")
f12a.ll<-drm(vivos/total~conc, droga, data=fin12,fct = LL.4(), weights = total, type=("binomial"))

op <- par(mfrow = c(1, 1))
plot(f1a.ll, broken = F, type = "all", main="Farm 1", xlab = "concentrations (%)",
     ylab = "resistance (x*100=%)", col=c("red", "black","blue"),
      cex.legend =  0.7, lty=1, lwd=1, xlim = range(Predic$conc), ylim = c(0,(1)))
```

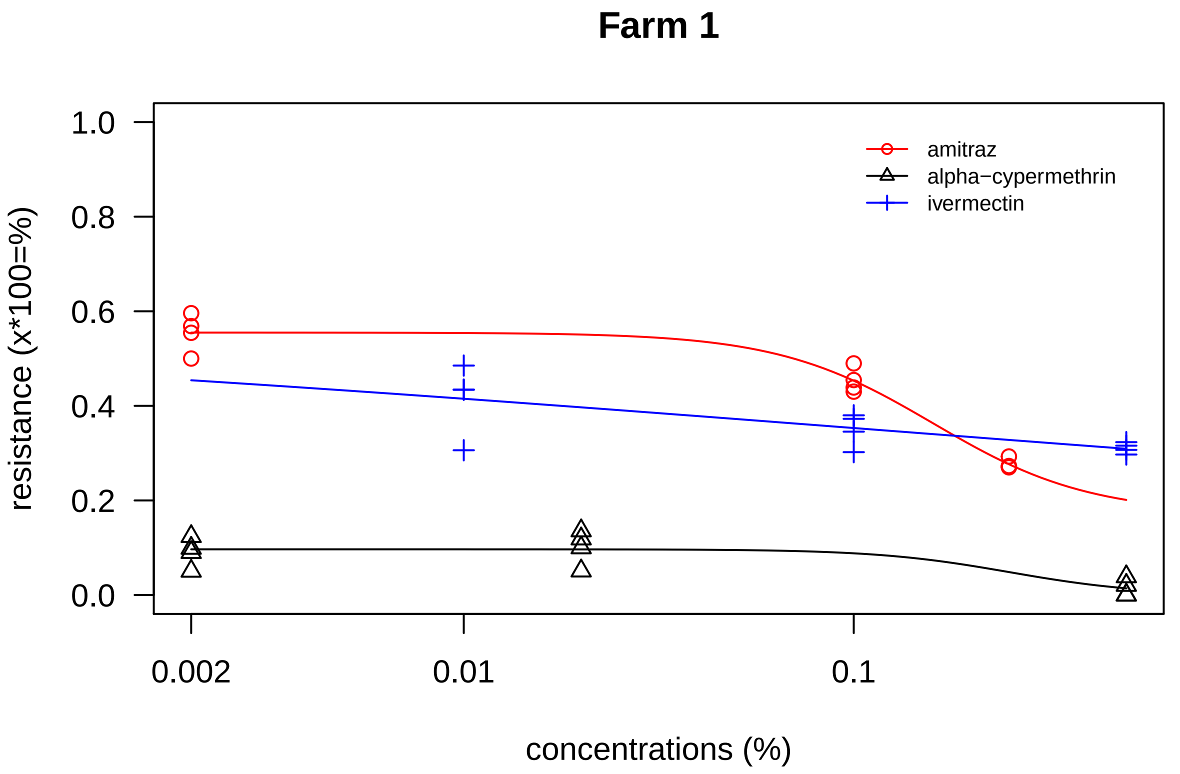


```
plot(f2a.ll,broken = T,type = "all", main="Farm 2", xlab = "concentrations (%)",
     xlim = range(Predic$conc), ylab = "resistance (x*100=%)", col=c("red", "black","blue"),
       cex.legend =   0.7, lty=1, lwd=1)
```

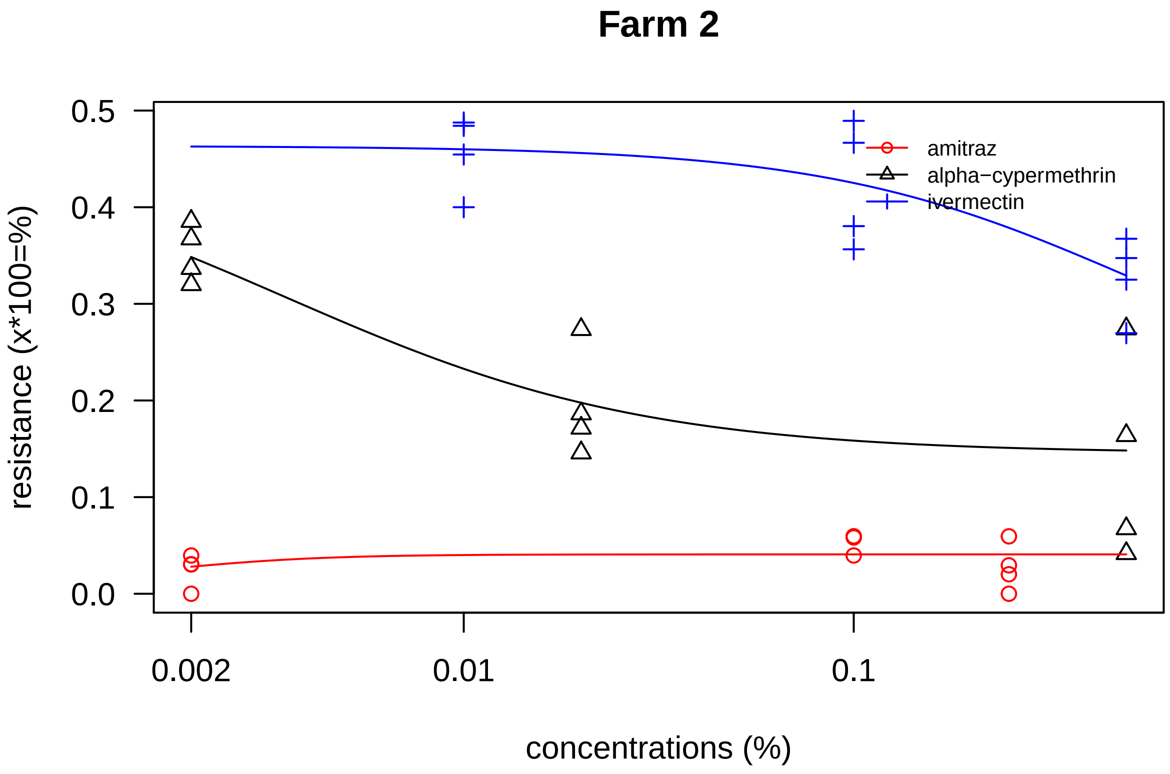


```
plot(f3a.ll,broken = T,type = "all", main="Farm 3",xlab = "concentrations (%)",
     xlim = range(Predic$conc), ylab = "resistance (x*100=%)", col=c("red", "black","blue"),
       cex.legend =  0.7, lty=1, lwd=1)
```

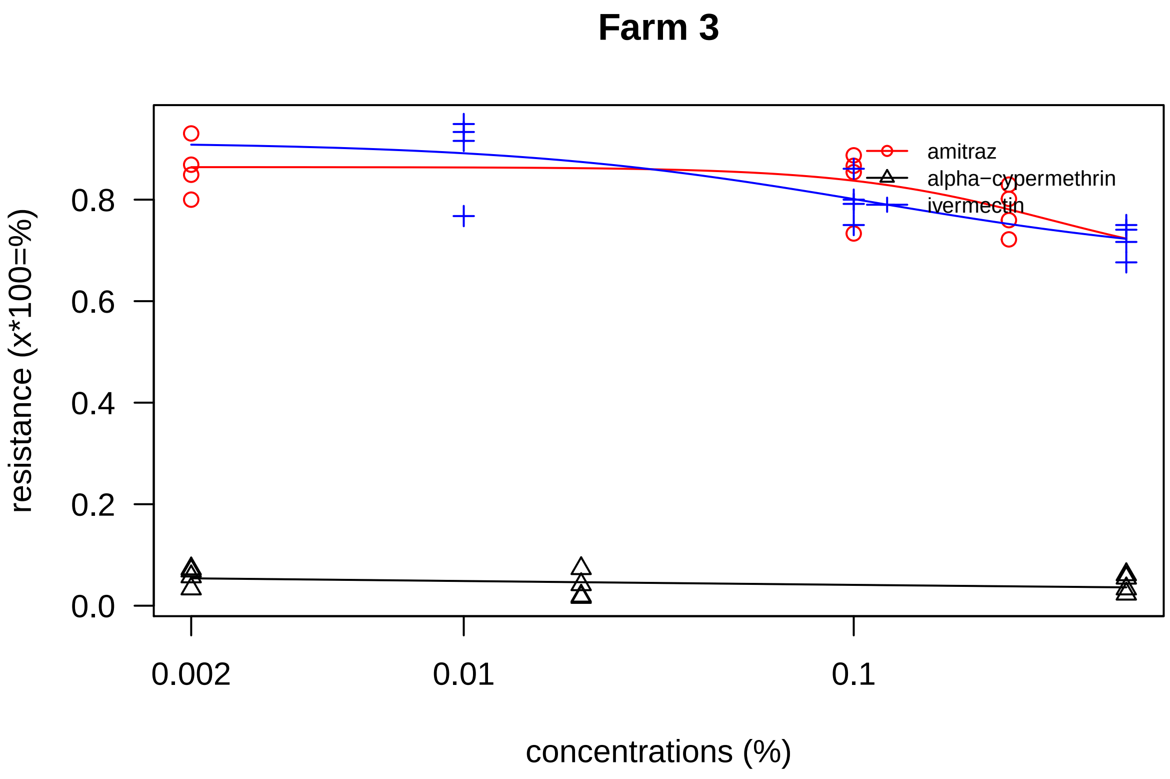

```
plot(f4a.ll,broken = T,type = "all", main="Farm 4",xlab = "concentrations (%)",
     xlim = range(Predic$conc), ylab = "resistance (x*100=%)", col=c("red", "black","blue"),
       cex.legend =  0.7, lty=1, lwd=1)
```

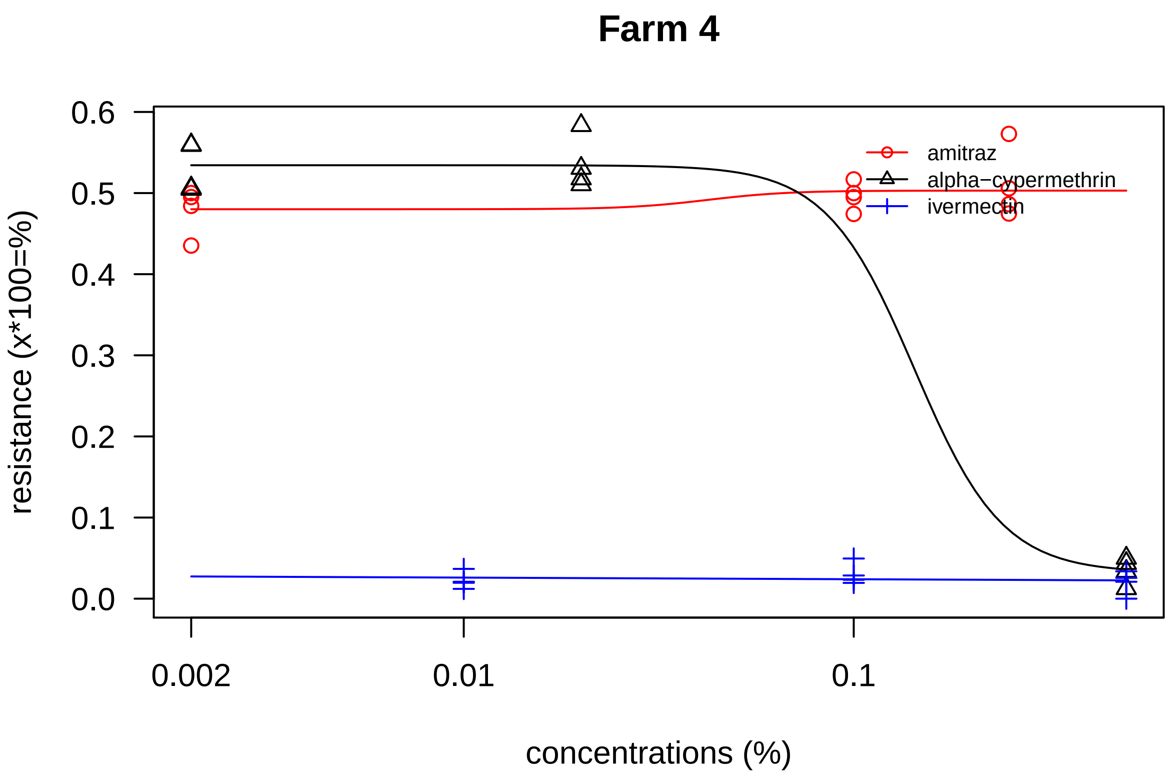


```
plot(f5a.ll,broken = T,type = "all", main="Farm 5",xlab = "concentrations (%)",
     xlim = range(Predic$conc), ylab = "resistance (x*100=%)", col=c("red", "black","blue"),
       cex.legend =  0.7, lty=1, lwd=1)
```

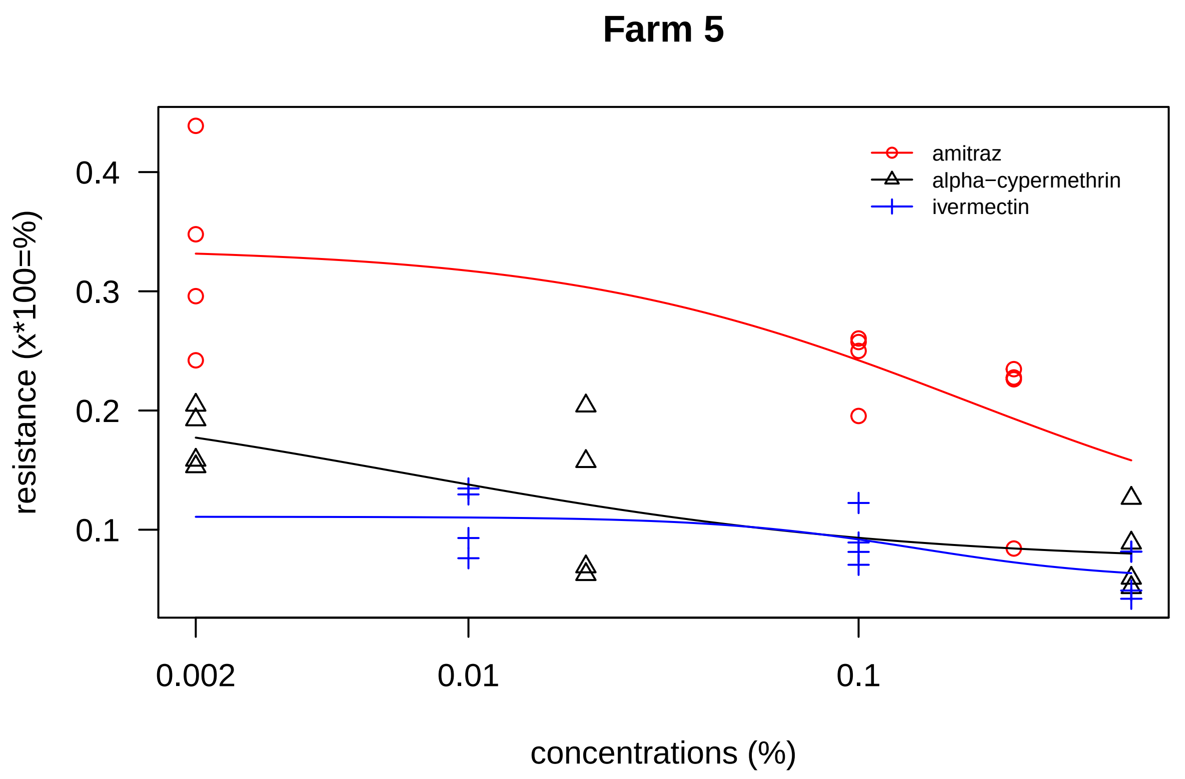


```r
plot(f6a.ll,broken = T,type = "all", main="Farm 6",xlab = "concentrations (%)",
     xlim = range(Predic$conc), ylab = "resistance (x*100=%)", col=c("red", "black","blue"),
       cex.legend =  0.7, lty=1, lwd=1)
```

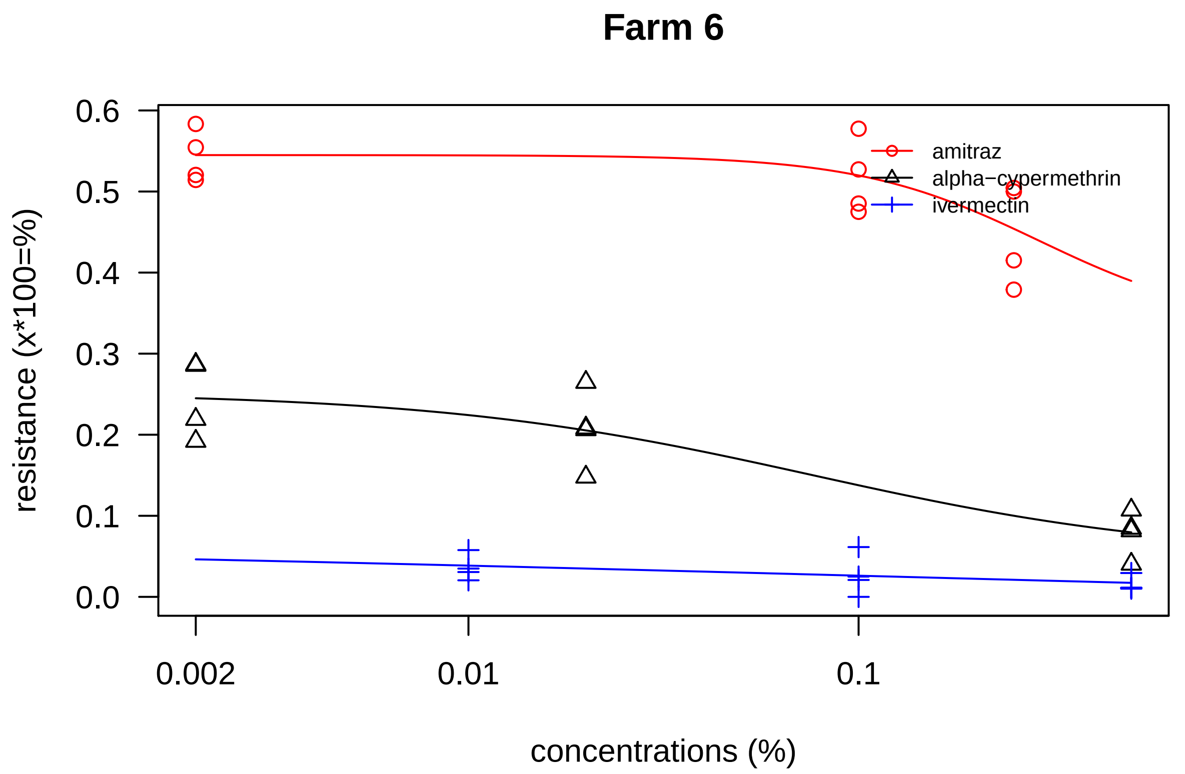

```
plot(f7a.ll,broken = T,type = "all", main="Farm 7", xlab = "concentrations (%)",
     xlim = range(Predic$conc), ylab = "resistance (x*100=%)", col=c("red", "black","blue"),
       cex.legend =  0.7, lty=1, lwd=1)
```

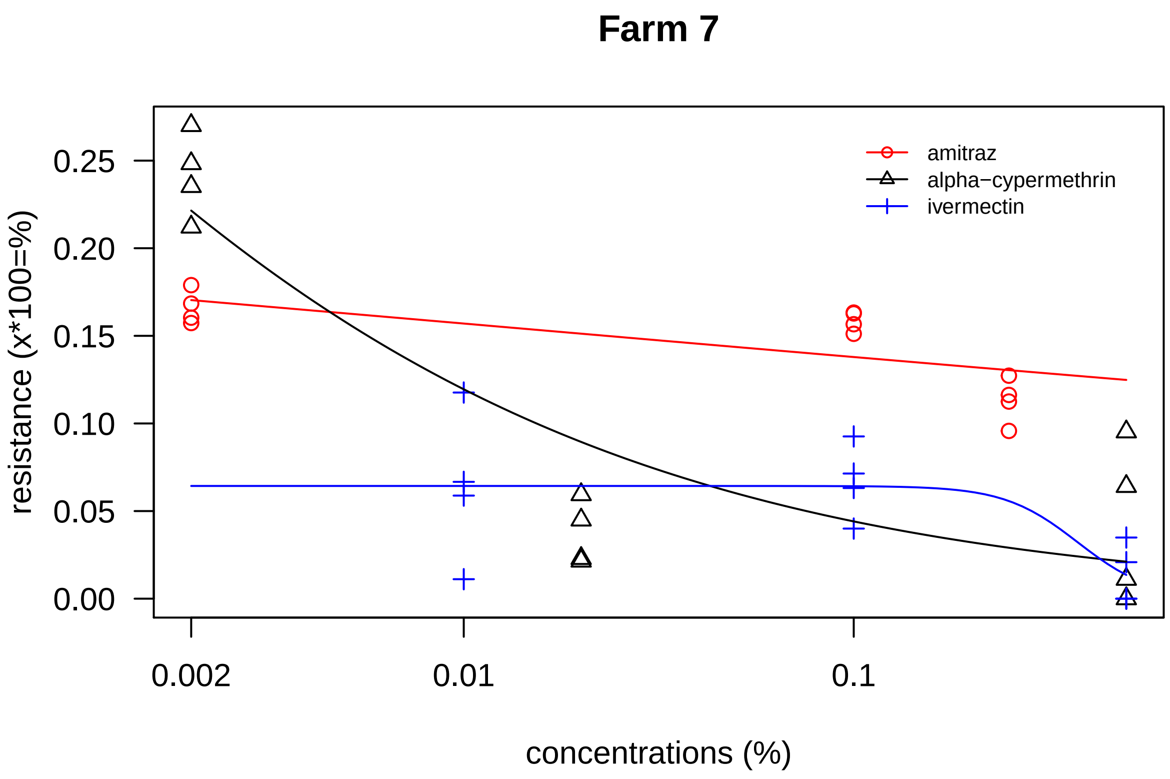


```
plot(f8a.ll,broken = T,type = "all", main="Farm 8",xlab = "concentrations (%)",
     xlim = range(Predic$conc), ylab = "resistance (x*100=%)", col=c("red", "black","blue"),
       cex.legend =  0.7, lty=1, lwd=1)
```

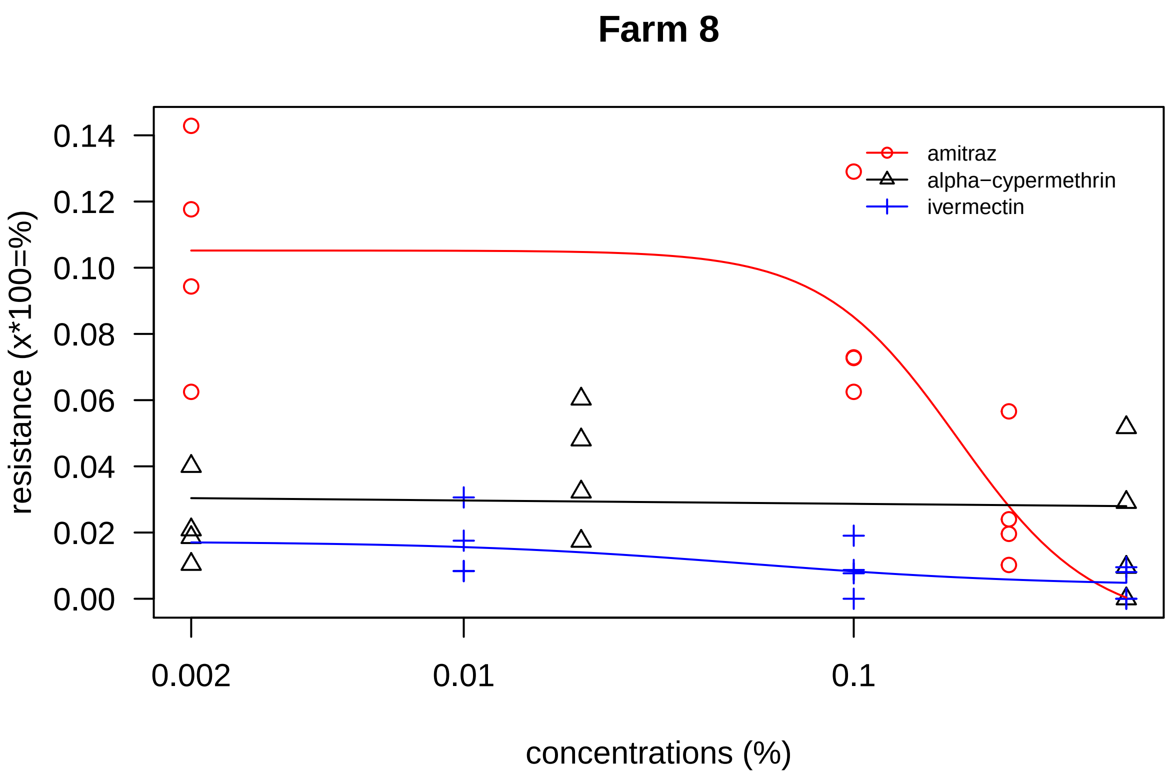


```
plot(f9a.ll,broken = T,type = "all", main="Farm 9",xlab = "concentrations (%)",
     xlim = range(Predic$conc), ylab = "resistance (x*100=%)", col=c("red", "black","blue"),
       cex.legend =  0.7, lty=1, lwd=1)
```

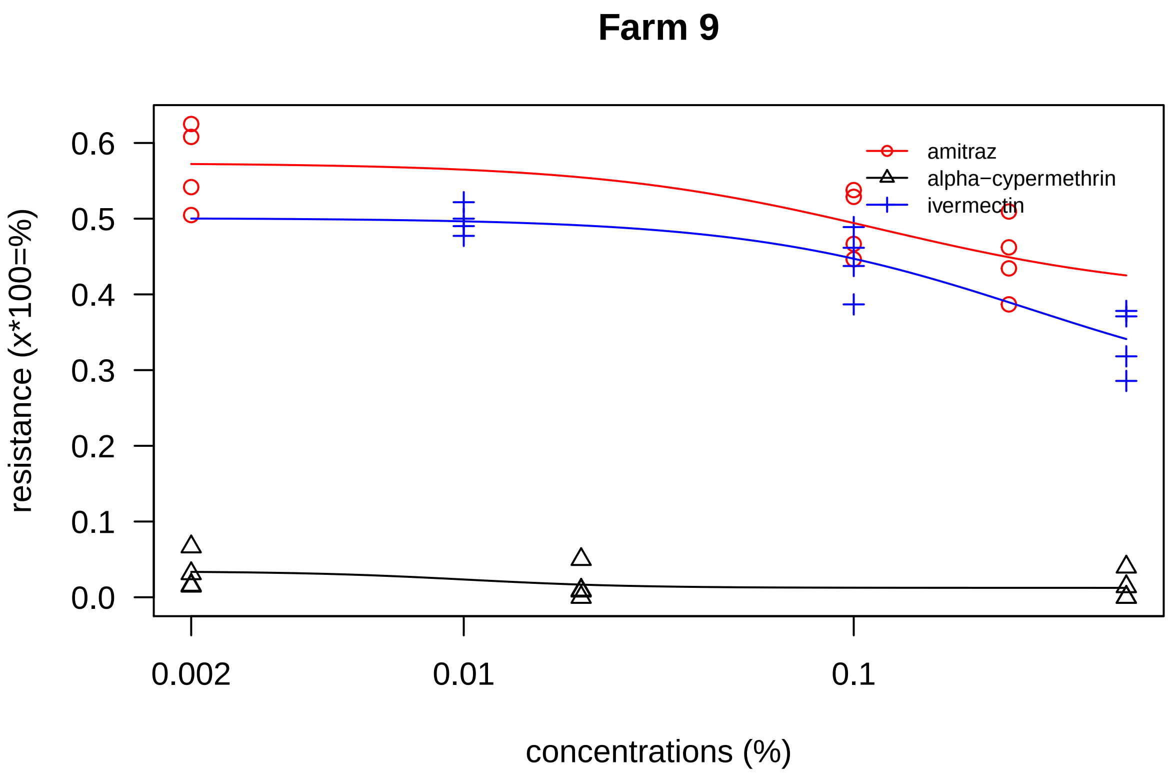

```r
plot(f10a.ll,broken = T,type = "all", main="Farm 10",xlab = "concentrations (%)",
     xlim = range(Predic$conc), ylab = "resistance (x*100=%)", col=c("red", "black","blue"),
       cex.legend =  0.7, lty=1, lwd=1)
```

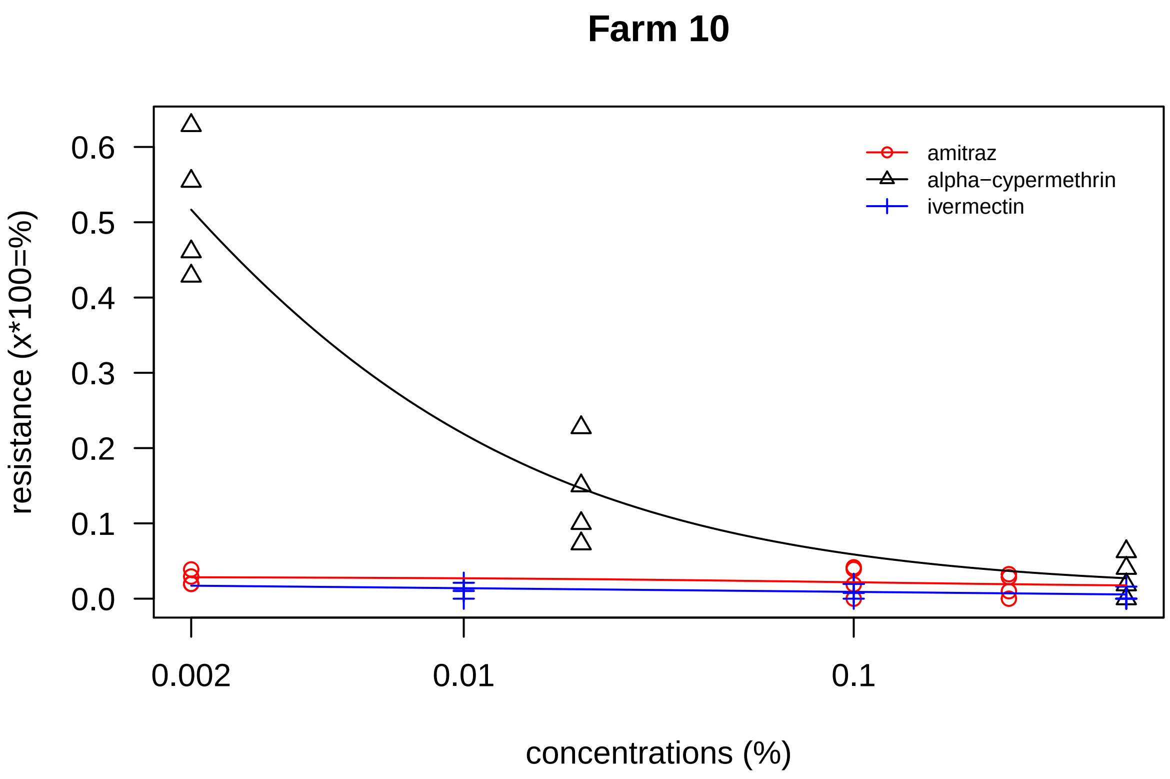


```r
plot(f11a.ll,broken = T,type = "all", main="Farm 11",xlab = "concentrations (%)",
     xlim = range(Predic$conc), ylab = "resistance (x*100=%)", col=c("red", "black","blue"),
       cex.legend =  0.7, lty=1, lwd=1)
```

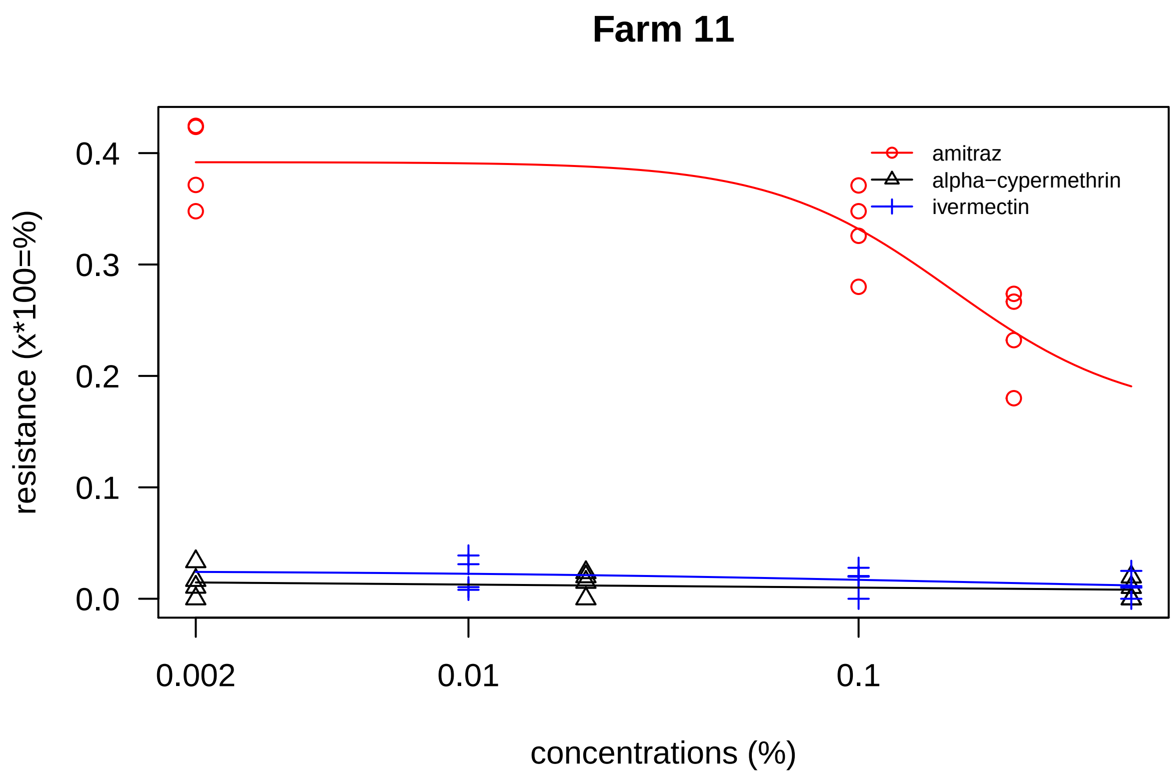


```
plot(f12a.ll,broken = T,type = "all", main="Farm 12",xlab = "concentrations (%)",
     xlim = range(Predic$conc), ylab = "resistance (x*100=%)", col=c("red", "black","blue"),
       cex.legend =  0.7, lty=1, lwd=1)
```

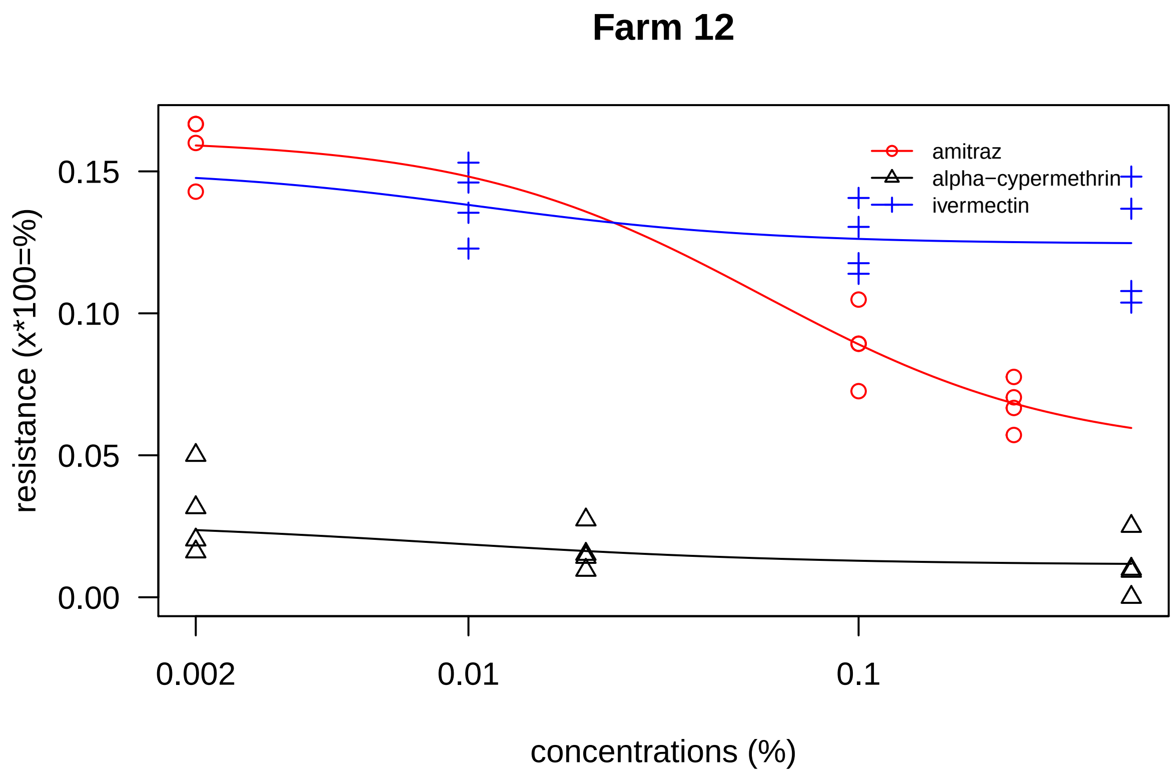

Supplement: S2 R Analysis — Analysis were based on three different concentrations per acaricide i.e. alpha-cypermethrin at 0.002%, 0.02%, and 0.5%; amitraz at 0.002%, 0.1%, and 0.25% and ivermectin at 0.01%, 0.1%, and 0.5% for the minimum, medium or discriminatory and maximum doses, respectively. All farms except farm 8 showed resistance levels (more than 10%) according to use frequency of each acaricide (PDF) [file pone.0174652.s002.pdf]
